# Geochemical properties of blue carbon sediments through an elevation gradient: study of an anthropogenically impacted coastal lagoon

**DOI:** 10.1007/s10533-022-00974-0

**Published:** 2023-02-08

**Authors:** Anthony Grey, Ricardo Costeira, Emmaline Lorenzo, Sean O’Kane, Margaret V. McCaul, Tim McCarthy, Sean F. Jordan, Christopher C. R. Allen, Brian P. Kelleher

**Affiliations:** 1 School of Chemical Sciences, Dublin City University, Glasnevin, Dublin 9, Ireland; 2 The School of Biological Sciences, Queen’s University Belfast, Belfast, N. Ireland; 3 Department of Chemistry, University of Kansas, Lawrence, KS 66045 USA; 4 National Centre for Geocomputation, Maynooth University, Kildare, Ireland; 5 Insight SFI Research Centre for Data Analytics, Dublin City University, Dublin, Ireland

**Keywords:** Blue carbon, Saltmarsh, Geochemical properties, Elevation gradient, Anthropogenically impacte

## Abstract

Global research is showing that coastal blue carbon ecosystems are vulnerable to climate change driven threats including accelerated sea-level rise and prolonged periods of drought. Furthermore, direct anthropogenic impacts present immediate threats through deterioration of coastal water quality, land reclamation, long-term impact to sediment biogeochemical cycling. These threats will invariably alter the future efficacy of carbon (C) sequestration processes and it is imperative that currently existing blue carbon habitats be protected. Knowledge of underlying biogeochemical, physical and hydrological interactions occurring in functioning blue carbon habitats is essential for developing strategies to mitigate threats, and promote conditions to optimise C sequestration/storage. In this current work, we investigated how sediment geochemistry (0–10 cm depth) responds to elevation, an edaphic factor driven by long-term hydrological regimes consequently exerting control over particle sedimentation rates and vegetation succession. This study was performed in an anthropogenically impacted blue carbon habitat along a coastal ecotone encompassing an elevation gradient transect from intertidal sediments (un-vegetated and covered daily by tidal water), through vegetated salt marsh sediments (periodically covered by spring tides and flooding events), on Bull Island, Dublin Bay. We determined the quantity and distributions of bulk geochemical characteristics in sediments through the elevation gradient, including total organic carbon (TOC), total nitrogen (TN), total metals, silt, clay, and also, 16 individual polyaromatic hydrocarbon’s (PAH’s) as an indication of anthropogenic input. Elevation measurements for sample sites were determined on this gradient using a LiDAR scanner accompanied by an IGI inertial measurement unit (IMU) on board a light aircraft. Considering the gradient from the Tidal mud zone (T), through the low-mid marsh (M) to the most elevated upper marsh (H), there were significant differences between all zones for many measured environmental variables. The results of significance testing using Kruskal–Wallis analysis revealed, that %C, %N, PAH (µg/g), Mn (mg/kg), TOC:NH_4_^+^ and pH are significantly different between all zones on the elevation gradient. The highest values for all these variables exists (excluding pH which followed a reverse trend) in zone H, decreasing in zone M and lowest in the un-vegetated zone T. TC content is 16 fold higher overall in vegetated (3.43 -21.84%) than uninhabited (0.21–0.56%) sediments. TN was over 50 times higher (0.24–1.76%), more specifically increasing in % mass on approach to the upper salt marsh with distance from the tidal flats sediments zone T (0.002–0.05%). Clay and silt distributions were greatest in vegetated sediments, increasing in % content towards upper marsh zones The retention of water, metals, PAHs, mud, chloride ions, NH_4_^+^, PO_4_^3−^ and SO_4_^2−^ increased with elevated C concentrations, concurrently where pH significantly decreased. Sediments were categorized with respect to PAH contamination where all SM samples were placed in the high polluted category. The results highlight the ability of Blue C sediments to immobilise increasing levels of C, N, and metals, and PAH with over time and with both lateral and vertical expansion. This study provides a valuable data set for an anthropogenically impacted blue carbon habitat predicted to suffer from sea-level rise and exponential urban development.

**Graphical abstract:**

Summarized results from this study demonstrating the geochemical changes through an elevation gradient, with a transect encompassing intertidal sediments through supratidal salt marsh sediments within Bull Island’s blue carbon lagoon zones.

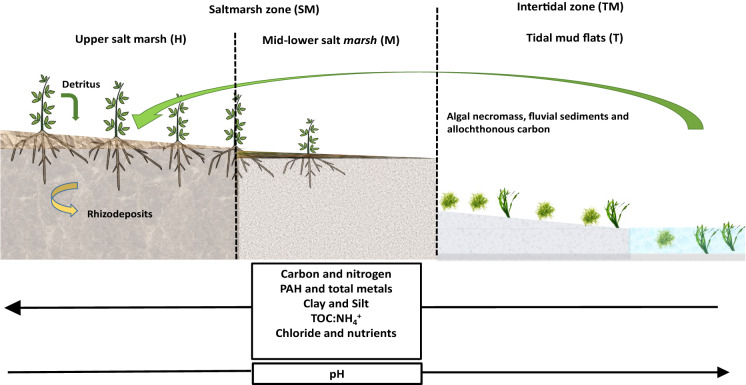

**Supplementary Information:**

The online version contains supplementary material available at 10.1007/s10533-022-00974-0.

## Introduction

Coastal wetlands are heterogeneous systems of great biodiversity and are one of the world’s most important carbon sinks, globally recognised by the term ‘Blue carbon’ ecosystems (Chmura et al. 2003; Nellemann et al. 2009; McLeod et al. 2011). Blue carbon is a term applied to C capture in marine based environments, encompassing autochthonous C from marine based organisms and burial of allochthonous C in marine sediments, generally transported as fluvial materials by riverine systems (Regnier et al. 2013; Kelleway et al. 2017; Kumar et al. 2020; Grey et al. 2021). These habitats are estimated to capture between 235 and 450 Tg C every year—or the equivalent of up to half of the emissions from the entire global transport sector, estimated at around 1000 TgC yr^−1^ (Nellemann et al. 2009). There is growing evidence and consensus that promoting the conservation, restoration, and sustainable use of coastal ecosystems has great potential as an effective tool in global carbon management and offsetting atmospheric gas emissions.

Coastal wetlands present great habitat diversity where natural ecotones form between subtidal, intertidal and supratidal sediments, driven by variations in hydrological regimes, elevation gradients and vegetation succession (Macreadie et al. 2017). Transition from intertidal to supratidal vegetated coastal ecosystems (VCEs) represents a transition to higher states of potential C accumulation as fresh OM inputs arise from plant biomass both above and below ground (Mudd et al. 2009; McLeod et al. 2011; Fagherazzi et al. 2012; Gunnell et al. 2013; Angst et al. 2018). Additionally, succession of vegetation provides a conduit for C exchange between the atmosphere, the sediments and microorganisms, as captured CO_2_ is converted into oxygen, and labile rhizodeposits in conjunction with more recalcitrant detritus. Furthermore, coastal wetland sediments provide a blueprint for constructed wetlands by demonstrating ability to operate as coastal filters through capture, immobilisation and detoxification of heavy metals (e.g. lead and zinc) (Stumpner et al. 2018), organic pollutants (e.g. Polyaromatic Hydrocarbons (PAH)) (Cottin and Merlin 2008) and excessive nutrients (nitrogen and phosphorus) (Vymazal 2007), especially in vegetated coastal ecosystems (VCE). Indeed, PAHs and metals have been shown to have high affinity for attachment to both OC and fluvial particles (i.e. clay and silt) in marine environments (Kleber et al. 2007; Kleber et al. 2015; Gregg et al. 2015). This relationship can begin with coexistence at source and through abiotic or biotic catalysed interactions during transportation and sedimentation (Duran and Cravo-Laureau 2016; Gonçalves et al. 2016). The redox dynamics associated with biogeochemical cycling in the rhizosphere horizon of sediment enhances metal accumulation around plant roots, which is the zone that accounts for the majority of plant–microbe interactions (Spivak et al. 2019). The rhizosphere synergy of plant-sediment-microbial interactions is a primary driver of C accumulation in water-logged vegetated coastal ecosystems where fluctuating redox conditions and potentially high anthropogenic impacts present dynamic metabolic challenges within the rhizosphere horizons (Ortíz-Castro et al. 2009; Jacoby et al. 2017; Barré et al. 2018). Ultimately, C and associated sediment constituents in such settings can accumulate for centuries but will be very much influenced by processes such as sea level change, ocean acidification, increased flooding, periods of drought and anthropogenic contributions (Castillo et al. 2000). Future restoration, coastal protection and carbon sequestration schemes will need to consider coastal sediment characteristics and their potential to capture, cycle and sequester both marine and terrestrial derived materials. A model site which will help in this regard is Bull Island (BI), a functioning blue carbon habitat performing as a coastal filter in Dublin Bay, Ireland (Grey et al. 2021).

**Fig. 1:**
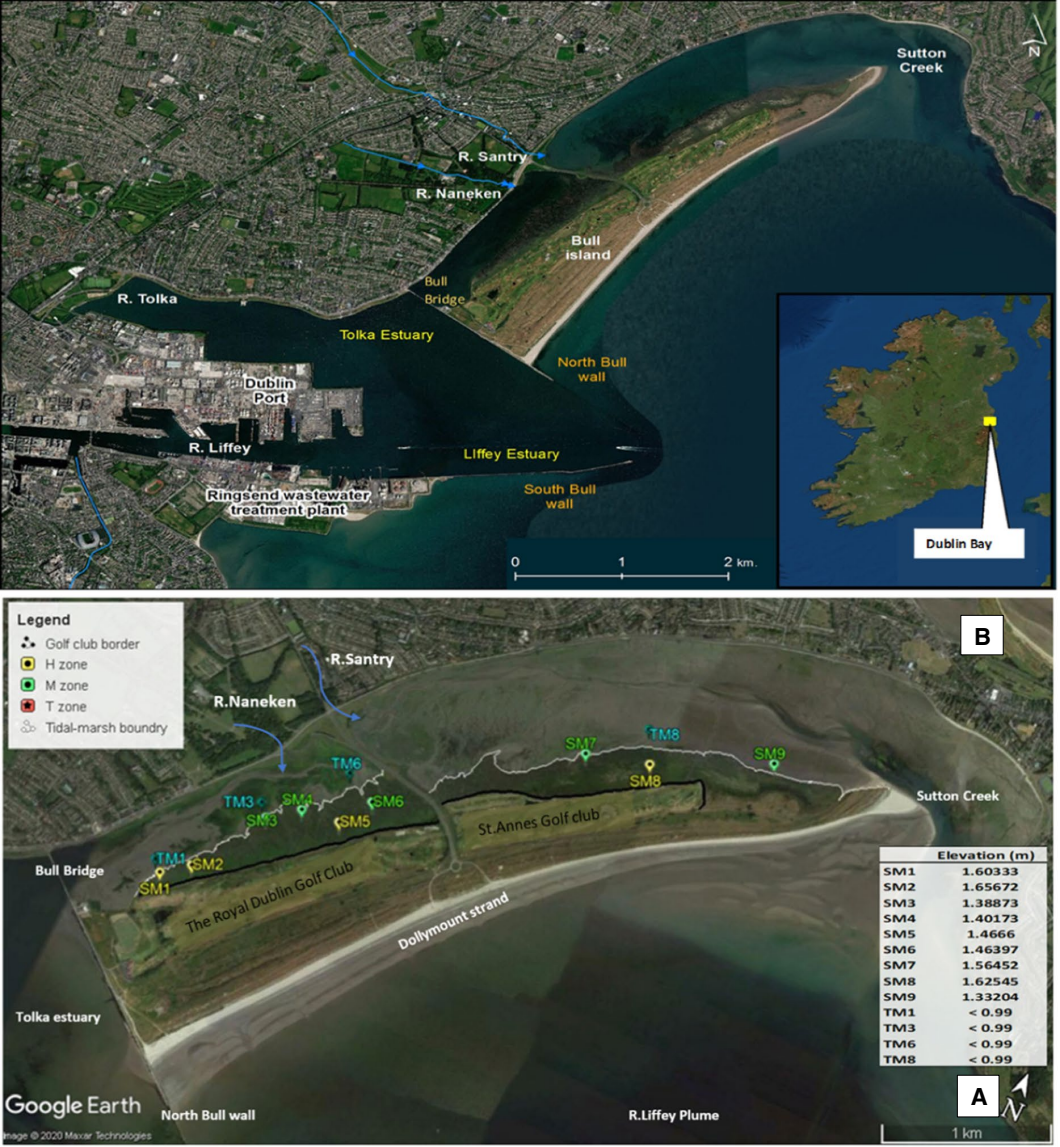
**A** Map of Bull island catchment area with surrounding urbanised areas indicated. **B** Overview of Bull Island study area (53.3705◦ N, 6.1440◦ W) and the individual sampling points. Defined sample zones are colour coded as described in the legend: Intertidal zone T = blue, Supratidal mid-Lower marsh zone M = green and Supratidal upper marsh zone H = yellow. Datum elevation of sample sites is reported in meters (m) as calculated using Lidar. Tidal sample points TM3 and TM6 were outside the Lidar survey therefore are reported as less than the minimum tidal elevation point measured i.e. < 0.74 m. All site GPS coordinates are listed in Table 1

**Fig. 2:**
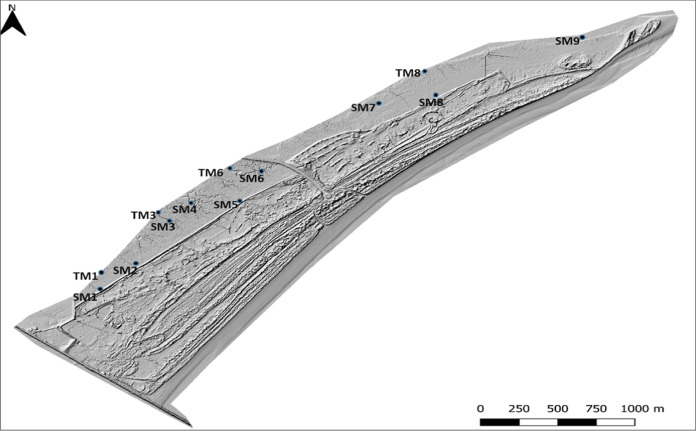
LiDAR DTM Bull Island displayed as hill-shade image with sample sites included

BI (53.3705° N, 6.1440° W) is a coastal sand spit expanding 5 km northeastwards from the north wall of Dublin Port and 800 m in breadth at its widest point. It formed as an unintended consequence of the construction of north and south Bull walls, built over 200 years ago in Dublin Port, to alleviate silting of the shipping route. Tidal changes induced by the construction of the walls resulted in the deposition of sand and silt in the north inner bay and an actively accreting dune system that continues to this day (National Parks and Wildlife Service 2014; Mathew et al. 2019). Two adjacent but unconnected lagoons exist on the landside of BI, the south lagoon (SL) and the north lagoon (NL). Both lagoons possess vegetated saltmarsh (SM) and un-vegetated mudflats (MF) wetland sediments. The lagoons are fed by different freshwater and tidal sources (Fig. 2a), with the SL directly connected to the Tolka estuary and Naniken Brook catchments., while the NL receives mixed marine waters passing into Sutton creek through the R. Liffey plume and the Santry River. Nutrient input into the BI lagoons has been a major problem in the past, with the main sources including input from rivers (Wilson 2005), raw sewage and industrial pollution (Jeffrey and Hayes 2005; Bedri et al. 2006; Murphy et al. 2016). BI is adjacent to Dublin Port (Fig. 1), the busiest port in Ireland, which contains several other industries including a wastewater treatment plant and a waste-to-energy incinerator. The Bull lagoons have consistently been identified as the areas that have been most impacted by pollution (Jeffrey et al. 1978; Choiseul et al. 1998; Jeffrey and Hayes 2005; Buggy 2006). Particulates from sewage and rivers have been identified as a major reason for both the productivity and the structuring of the food web in Dublin Bay, and are a major influence of the energy budget for the bay (Wilson et al. 2002). BI’s tidal wetland zones shares the capability of coastal wetlands to sequester carbon (blue carbon), immobilize and treat anthropogenic and natural contamination, create wildlife habitats and provide protection from the looming threats of sea-level rise (SLR) (Devoy 2008). Recent work by Nejad et al.(2022) has provided an updated sea level dataset for Dublin for the period 1938–2016 at yearly resolution (Nejad et al. 2022). The authors documented Dublin Bay to have an estimated rate of SLR of 1.1 mm yr^−1^ during 1953–2016 (95% credible interval from 0.6 to 1.6 mm yr^−1^), and a rate of 7 mm yr^−1^ during 1997–2016 (95% credible interval from 5 to 8.8 mm yr^−1^). This research highlights the future threats facing such urbanized coastal zones, especially as historical estimates have large multi-decadal variability in data sets, which hides the actual higher rates of SLR in recent years. A previous geochemical study by Grey et al. (2021) measured, mapped and reported sediment properties for 59 sample locations covering representative zones across all of BI (Grey et al. 2021). In the context of blue carbon sediments, the authors showed the differences in the bulk geochemical characteristics between SM and MF sediments, and in NL and SL sediments. The establishment of BI as a blue carbon capture zone is a direct result of anthropogenic diversion of natural processes, thereafter, driven by hydrologically constrained distribution of process outputs and succession of resilient biota.

This current study was performed along a coastal ecotone encompassing an established elevation gradient transect from intertidal sediments (un-vegetated and covered daily by tidal water), through vegetated salt marsh sediments (supratidal-periodically covered by spring tides and flooding events), on Bull Island, Dublin Bay. Similarly to Grey et al.(2021), we hypothesised that the distribution of OM would align with PAH and bulk metals including Fe, Pb, Zn, Mn and P, and that concentrations would be higher in vegetated salt marsh sediments as elevation increased, thus expected most metals distributions to be controlled by OM concentrations. A higher fluvial sediment content was expected in vegetated marsh due to dissipation of water energy by facilitating finer particle deposition i.e. clay and silt. We aimed to investigate changes in sediment geochemistry and relationships of variables to elevation of sample sites in order to evaluate the influence of OM on metal and PAH distributions with accretion. We attained elevation measurements using a combination of LiDAR measurements, digital terrain models (DTM) and digital surface models (DSM). Edaphic variables measured included C, N, PAH, nutrient concentrations, total metals, and total PAH, including distributions partitioned by ring structure groups in sediment zones.

We used Spearman’s correlation analysis, PCA and Kruskal–Wallis testing for significance differences between group variables to test our questions. We aimed to use physical and geochemical signatures to investigate changes in sediment across an elevation gradient that indicated exposure to different hydrological conditions. The results of this multidisciplinary study are important in the context of coastal wetland biogeochemistry as we report on the sedimentary association of metals and pollutants with blue C accumulation on an elevation gradient, which represents different stages of habitat formation. Furthermore, the results highlight the ability of Blue C sediments to immobilise increasing levels of C, N, and metals, and PAH with over time and with both lateral and vertical expansion. Importantly, the emergence and functioning of these sedimentary habitats has evolved under the constraints of a complete history involving anthropogenic influences. Ultimately, this study presents a geochemical data set for a functioning but geologically young blue carbon zone, currently a C and pollutant sink, however, facing an imminent future threat from SLR.

### Study site description

The formation and continual advancing of Bull Island (53.3705° N, 6.1440° W) involves a combination of constant destruction and growth, dictated by the hydrological dynamics of Dublin Bay over approximately 300 years (Flood 1975). The growth of the island in the nineteenth century resulted in a sheltered, single shallow creek or lagoon between the island and the mainland (Academy 2020). Sheltered lagoon zones provide an area of energy dissipation for incoming tidal water, thus facilitating higher levels of deposition for suspended particles. Previously, tidal waters entering from the two ends of the island met between the mouths of the Naniken Brook and Santry River (Fig. 1).Indeed recently identified as two high risk waterbodies under the 3rd cycle of Water Framework Directive assessment [https://gis.epa.ie/EPAMaps/Water] (rpsgroup 2021). During this time, approximately 200 m’ breadth of vegetated saltmarsh emerged, driven by silt deposition coming mainly from the rivers. In 1964, a constructed causeway at the area where the tidal waters converged in the lagoon made the northern part of the island more accessible. This had the effect of making two independent lagoons, north lagoon (NL) and south lagoon (SL), zones analogous to artificially constructed wetlands, each with their own characteristics.

The estuary of the R. Liffey receives storm-water run-off from Dublin City, and treated wastewater from Ringsend wastewater treatment plant which performs primary, secondary and partial tertiary treatments (UV screening) of sewage from Dublin City (O’Boyle and Wayne 2017; Norton et al. 2018). The EPA have identified and reported waste water discharges as the sole significant pressure on water bodies at risk of pollution in the Dublin Bay catchment zone including, the estuarine systems and both Merrion strand and Sandymount beaches (EPA Ireland 2017). Prior to the construction of underwater pipelines from south Dublin bay at Dunlaoighre in 1991 and north Dublin bay at Howth in 2001, wastewater from these areas were discharged to the bay after only basic screening. Particulates from sewage and rivers have been identified as a major reason for both the productivity and the structuring of the food web in Dublin Bay, and are a major influence of the energy budget for the bay (Wilson et al. 2007). Nutrient input into the Bull island lagoons has been a major problem in the past, with the main sources including input from rivers (Wilson 2005), raw sewage and industrial pollution (Wilson et al. 2002; Jeffrey and Hayes 2005; O’Higgins and Wilson 2005; Murphy et al. 2016).

Intertidal zones on the south lagoon are un-vegetated. During spring, summer and autumn months, sediments become inhabited with large areas of algae blooms (Enteromorpha spp, Ulva Lactuca), seaweed, and patches of grey and muddy sand. The inner north lagoon has a pioneer marsh zone which gradually transitions into saltmarsh. The intertidal zone (Zone T) is covered 100% by tides (Healy 1975). Low cliffing at the marshes edge marks the phasing out of and the beginning of the gradient ascent into vegetated sediments classed as a saltmarsh (SM) habitat. The lower to mid reaches of the saltmarsh (Zone M) contains a highly inconsistent terrain with regions of *Spartina anglica* (common cordgrass), *Halimione portulacoides* (sea purslane), *Puccinellia maritima* (common salt marsh grass), and abundant but patchy *Salicornia L*, with both species *S. europea L*. and *S. dolichostachya* (glasswort) present in stages. Other flora includes *Plantago maritima, Armeria maritima, Triglochin maritima, Armeria maritima* and *Aster tripolium.* Channels scar the marshes, filling during tides to introduce saltwater into more porous substrata layers, Zone M is estimated to be 30–40% covered tides (Healy 1975). The upper salt marsh (Zone H) receives less tidal inundation and consequently has a more diverse flora. Many of the lower saltmarsh species occur but the upper salt marsh is characterised by *Juncus maritimus* and *Juncus gerardii* and a wider range of flora including *Glaux maritima, Triglochin palustris* and *Agrostis stolonifera*. Immediately below the underlying detritus layer, the sediments are moist, dark, and high in organic matter and penetrated throughout by living and decaying rhizosphere material. Zone H has been estimated to receive approximately 5 to 18% of tides (Healy 1975). Where tidal cover is reduced for prolonged periods, the influence of surface freshwater increases through rainfall, washing over the soil, thus mobilising ionic chemical species (Simpson et al. 2010; Gross and Harrison 2019), and subsurface with the subsequent rising of the groundwater table.

## Materials and methods

### Sampling

Sediments samples were extracted from Bull Island’s saltmarshes (SM) and intertidal mudflats (TM) habitats in both the north and south lagoons situated at the mainland side of the Island. Sampling for this study was carried out on the 15th and 16th of June 2017 with Met Eireann reporting 0 mm precipitation either day and max air temperatures of 18.9 and 21.7 °C respectively (https://www.met.ie/climate/available-data/historical-data). Samples were taken each day at low tide for a period when the intertidal sediments were fully exposed. Sample site observations were arbitrarily grouped according to sample location, relative to proximity golf courses (Fig. 1B), thus distance from the incoming tides. Groups were assigned in the following format:

High Saltmarsh (zone H): SM1, SM2, SM5 and SM8.

Mid-Lower Saltmarsh (zone M): SM3, SM4, SM6, SM7 and SM9.

Intertidal mud (zone T): TM1, TM3, TM6 and TM8.

Sample sites were selected to represent defined zones across an elevation gradient. The sediment conditions should hypothetically differ across a gradient of high marsh to mid-low marsh to tidal mud as the frequency of tidal inundation lessens i.e. towards the golf club. The distribution of sites was chosen in order to follow the deposition route of material transported to higher sediment areas following historical periods of tidal inundation. Sediment samples were taken to represent intertidal zone T, thereafter identified with the prefix ‘TM’ and a number corresponding to a sample site location as described by Table 1 e.g. TM3 (Table 1). Similarly, sediments in both lower/mid marsh zone M and upper marsh zone H identified with the prefix ‘SM’ and a number e.g. SM3 and SM8 respectively (Table 1). The assignment of two sample zones on salt marsh sediments were chosen to further resolve an elevation gradient and explore geochemical variations in the context of different degrees of tidal inundation following establishment of vegetation succession (Fig. 1B displays sample sites on Bull Islands lagoons).

**Table 1:** Displays sample site labels, GPS coordinates and sample site elevations (DTM meters) as determined by LiDAR

Lagoon	Zone	Sample Site	Latitude	Longitude	**LiDAR DTM Elevation (meters)**
South	H	SM1	53.361519	− 6.171543	1.6033
South	H	SM2	53.362581	− 6.169205	1.6567
South	M	SM3	53.366667	− 6.165014	1.3887
South	M	SM4	53.367862	− 6.162385	1.4017
South	H	SM5	53.368047	− 6.158798	1.4666
South	M	SM6	53.369822	− 6.156843	1.4639
North	M	SM7	53.377123	− 6.141078	1.5645
North	H	SM8	53.378	− 6.135333	1.6255
North	M	SM9	53.380836	− 6.125037	1.3320
South	T	TM1	53.362181	− 6.172519	0.9382
South	T	TM3	53.367431	− 6.166069	< 0.7400
South	T	TM6	53.3709	− 6.15995	< 0.7400
North	T	TM8	53.379911	− 6.13684	0.74401

At each sample site, a block of sediment (54,000 cm^3^ at covering approximately 12 cm depth) was removed intact from the ground using a clean stainless steel spade. Samples were immediately wrapped in furnaced aluminium foil and flash frozen onsite with liquid N_2_, to preserve biogeochemical integrity. Samples were bagged and returned to the lab for storage at -80ºC prior to subsampling. For geochemical and physical parameter analysis three ~ 200 g subsamples were taken at separate locations from the block, thereafter treated as individual samples (e.g. SM1 a, b and c) from each defrosted sediment block, encompassing a depth range from 0 to 10 cm after removal of loose surface detritus. Analytical replications (duplicate or triplicate) were performed in respective analysis for each of the, a, b and c samples at each site. This method was applied to all sample sites to account for heterogeneity of these sediment types (Bowen et al. 2009). Each subsample portion was screened by hand picking out roots, while carefully removing attached sediment particles and adding to the bulk sediment subsample. Screened sediments were sieved (2 mm mesh size) and mixed to enhance homogenisation of these inherently heterogeneous type sediments. Each sediment sample was then stored separately in sample bags at 4°c prior to further partitioning of samples for each separate chemical and physical parameters.

### LiDAR data capture and processing methodology for determination

The LiDAR data utilised here was captured on two separate dates in August 2017 and July 2018 by collaborators at Maynooth University, using the Riegel VUX-1LR LiDAR scanner accompanied by an IGI inertial measurement unit (IMU). The system was flown on board a Cessna 172 light aircraft using a wing strut mounted sensor pod. The aircraft flew at approximately 1000ft above ground level and overlap was achieved between flight lines. The resultant point density was approximately 5 points per meter, with a Z elevation accuracy of approximately 3 cm. After capture, the raw LiDAR and IMU trajectory data is processed. This essentially involves using a suite of software packages to combine the raw LiDAR data, raw trajectory data, and Ordinance Survey Ireland (OSI) GPS base station data to create a georeferenced LiDAR point cloud. The point cloud is outputted in the Irish Transvers Mercator coordinate system (EPSG 2157) [https://epsg.io/2157] with the OSGM15 geoid model. The post-processing steps involve cleaning, applying corrections to, and classifying the LiDAR point cloud. Firstly, the point cloud is inspected, and any erroneous/less than ideal points are manually removed (for example: points representing birds in flight, points captured at extreme scan angels, and points captured while the aircraft is banking). Next, tile line corrections were applied. These corrections correct for small Z (elevation) mismatches between flight lines. Finally, the point cloud is classified into pre-determined classes (for example: ground, buildings, vegetation). From the resultant LiDAR point cloud, digital terrain models (DTM) and digital surface models (DSM) were created at 1-m spatial resolution. A DTM represents the elevation values of bare ground, while a DSM represents the elevation values of the ground as well as all objects on the surface (for example: trees and buildings). Figure 2 shows an example of one such surface; a DTM of Bull Island represented as a hill-shade image. These modelled elevation surfaces were used to acquire elevation values at the locations of key sites. Table 1 displays results for elevation heights derived by LiDAR.

### Sediment geochemistry

#### % Organic Matter content in sediments (OM)

Prior to analysis, sediments were screened to remove larger structural plant debris and loosen attached soil particles. % SOM was determined through loss on ignition (Ball 1964). Analyses were performed in triplicate on 3–5 g samples (previously dried from % M determination) for each site. While a larger sample size of up to 20 g is known to improve precision of results, available sample was limited for this study (Hoogsteen et al. 2015). Samples were sieved to > 2 mm and ground using a mortar and pestle. They were then weighed (± 0.01 g) into ceramic crucibles, pre-weighed and tared on an analytical balance, covered with aluminium foil and a distinctive pattern pierced in each crucible for sample recognition. Crucibles were placed in a muffle furnace programmed to run at 550 °C for 8 h with a ramp rate of 10 °C /min^−1^. When cooled to room temperature, crucibles were removed and weighed on the same balance while containing the combusted soil residue. The mass of the crucible was subtracted from the total mass of the crucible/soil residue to obtain a mass of remaining inorganic compounds. The difference between inorganic and original mass, calculated as Δ mass was determined to be the mass of SOM lost during ignition, reported as a % of the original sample mass.

#### Sediment moisture content (%M), pH and Electrical conductivity (EC)

% M was calculated from the sample weight before and after drying. A quantity of each sediment was weighed out onto glass dishes and placed in an oven at 105 °C for a minimum of 24 h after which time a weight measurement was recorded. Vegetated salt marsh samples required 48 h of drying to achieve a stable dry mass due to higher detritus contents. Soil pH and EC were measured using the 1:5 method (soil:DiH2O w/v) (Rayment and Higginson 1992), a Cyberscan PC300 series pH/ meter (Eutech instruments) and a Thermo-scientific™ Orion star™ a222 conductivity portable meter. The pH probe was calibrated using buffer solutions at two points, pH 4.02 and pH 7.01 (Fisher Scientific, Dublin). All samples were analysed in duplicate using 1-3 g of wet soil in tubes with appropriate water volume and placed on a horizontal shaker at 150spm for 60 min. The pH probe was placed in the soil solution and briefly stirred. The measurement was recorded when the reading was stabilised and locked. The probe was rinsed repeatedly with DiH2O in between measurements. EC of soils was determined using the previously prepared sediment slurry after pH analysis. The conductivity meter was calibrated using a 12.65Ms/cm (12650 μS/cm) solution to accommodate the higher levels of salt present due to the marine nature of the sample site. The sediment extract solutions were filtered using a Buchner filtration apparatus (fisher scientific Glass fibre filters GF/A) and the conductivity probe was briefly stirred in the liquid filtrate. All EC measurements were standardised and recorded as μS/cm.

#### Sediment particle size analysis (PSA) and Elemental analysis

Physical sediment properties were analysed using % sand, % silt and % clay contents. The particle size of inorganic sediment particles was determined by laser granulometry using a Mastersizer 2000 particle size analyzer (Malvern, Worcestershire, UK) after removal of OM from sediment using a muffle furnace for 12 h at 380 °C. This was followed by sonication of remaining inorganic fraction in DI water to minimize aggregates interference (Vaasma 2008) and subsequent oven drying at 50 °C for 72 h. Sample analysis was carried out at the geography department in University College Cork. Elemental analysis was performed in triplicate on freeze-dried sediment samples using a Fisons NCS 1500 NA elemental combustion analyser to determine total carbon (%C), total nitrogen (%N) and total organic carbon (%TOC). The instrument was calibrated using an Acetanilide (C_8_H_9_NO) standard before every batch of samples and after every 9 samples thereafter. Blank runs were performed after triplicate sets to eliminate possible carry over due to high % OM content. For C and N analysis, freeze-dried, ground and sieved sediment samples (0.85 mm) were weighed into tin capsules, sealed and combusted in the presence of O_2_ to generated gaseous elements C and N, reported as % mass of initial sample. To determine % TOC content, freeze-dried samples were weighed and treated with 1 M hydrochloric acid in Ag capsules following the procedure of Verardo et al. (1990) to remove carbonate (Verardo et al. 1990), with subsequent measurements attained by combustion analysis. The original mass of the sample before HCL addition was used to attain the % TOC as a mass of the whole sample.

### X-ray fluorescence (XRF) analysis of metals

A portable Thermo Scientific Niton XL3t XRF instrument was used for the determination of metal concentrations in sediment samples. Metals analysed in this study included Iron (Fe), Sulfur (S), Zinc (Zn), Calcium (Ca), Aluminium (Al), Lead (Pb)Instrument performance and protocols were validated in previous studies (Radu and Diamond 2009; Radu et al. 2013). Sediment samples were screened, oven dried (105 °C for 48 h), ground with a mortar and pestle and sieved through a 0.85 mm sieve. Open-ended plastic sample tubes,32 mm in diameter and 50 mm deep were sealed at one end with a polypropylene X-ray film (Premier lab supply, FL, USA), and clipped into position with a plastic collar. The prepared sample was evenly deposited into the cup to cover the X-ray film, keeping the sample depth consistent at 5 mm. A cellulose disc was placed over the sample to hold contents, and polyester stuffing secured the disc in place. The cup was sealed with a plastic end cap. An internal instrument calibration was performed. An empty cup prepared as per sample was used for the blank. A solid heavy metal mix standard was used to check the accuracy of the instrument, where standard values determined from the Resource Conservation and Recovery Act (RCRA- U.S 1974) which provides guidance on waste disposal criteria. All measurements taken were performed in triplicate and analysed in bulk mode (spectral scan for all heavy metals). Each sample was analysed for 100 s.

### Polyaromatic hydrocarbon (PAH) extraction and analysis

Firstly, the 16 priority PAH (Naphthalene, Acenaphthylene, Acenaphthene, Fluorene, Phenanthrene, Anthracene, Fluoranthene, Pyrene, Chrysene, Benzo(a)anthracene, Benzo(k)Fluoranthene, Benzo(b)Fluoranthene, Benzo(a)pyrene, Indeno(1,2,3-c-d) pyrene, Dibenzo(a,h)anthracene, and Benzo(g,h,i) perylene.) pollutant concentrations and distributions were determined. Next, the mass (µg/g) and % contribution of PAH ring number groups (2–3, 4, 5 and 6 ring PAH to each sample site and zone were established. PAH results are discussed as a pollution index to investigate a geochemical aspect of sediments, discussed thereafter as a proxy for anthropogenic impacts (Silva et al. 2013; Pastene et al. 2019). We tested for significant differences and pairwise comparisons in concentrations of PAH, between group means for zones H, M and T. Furthermore, the same tests were performed for grouped SM (HT_sum_) samples (i.e. SM1-SM9, n = 27) vs grouped TM (i.e. TM1, TM3, TM6 and TM8, n = 12) samples to compare two sediment habitats facilitating strongly contrasting biogeochemical cycles. Additionally, the 2 lagoons were explored, NL (n = 12) Vs SL (n = 27) to compare adjacent but unconnected depositional zones which are historically (since the construction of a diving causeway in 1964) subjected to different hydrological regimes as previously reported (Grey et al. 2021). The value for the sum of 16 priority PAHs at individual sample sites and subsequent zones was used to assign a PAH pollutant level classification to sediments. Classification was applied as previously suggested by Baumard et al.(1998) where, PAH pollution classification was applied as follows: (a) low, 0–0.1 µg/g; (b) moderate, 0.1–1 µg/g; (c) high, 1–5 µg/g; and (d) very high, > 5 µg/g] (Baumard et al. 1998).

All sediment samples were air dried and extracted with heated dichloromethane (DCM) using a Dionex Accelerated Solvent Extractor (ASE) (ASE® 200 Accelerated Solvent Extractor) instrument (EPA method 3545A 2007) as described in previous work by Grey et al. (2021). DCM extracts were reduced down (~ 500 µl) at room temperature/low vacuum using rotary evaporation and made up to 1 ml in solvent washed volumetric flasks. The 1 ml extracts were transferred into pre-labelled GC vials and a spatula tip full of activated copper was added to the extracts to remove sulfur**.** Vials were shaken for 24 h in the dark, then subsampled and stored upright at − 30 °C until analysis by gas-chromatography mass spectrometry (GCMS). Analysis of extracts was carried out on an Agilent 7890 N gas chromatograph coupled to an Agilent 5973 N mass selective detector operating in electron impact mode at 70 eV. The data output was processed using Chemstation software, combining mass spectral library databases (NIST and Wiley), certified PAH standards, spectra interpretation, retention times and referenced literature to confirm presence of identified compounds. An internal standard of 100 mg/l 5α-cholestane was used for all extracts and blanks. 16 priority PAHs were quantified in SIM mode using the cholestane internal standard and a calibration curve produced from a 16 PAH certified reference material standard. The limit of quantification (LOQ) and limit of detection (LOD) was calculated for each group of PAH compounds according to the number of benzene rings. A LOQ and LOD was determined for 2 ring PAHs using naphthalene, fluorene (3- ring), phenanthrene (4-ring), pyrene (5-ring) and benzo (ghi) perylene (6-ring). The LOQ for each PAH ranged from 22.50 ng/g (all 5 and 6-ring PAHs) to 67.50 ng/g (fluorene). The LOD ranged from 0.0074 µg/g (all 5 and 6-ring PAHs) to 0.0222 µg/g (fluorene). A % recovery study was carried out using deuterated PAH compounds and the described ASE method (see supplementary data section for PAH extraction and analysis for further details on GCMS conditions and recovery study).

### Nutrient and anion analysis

For the analysis of water-soluble nutrients NH_4_^+^, NO_2_^−^, SO_4_^−2^ and PO_4_^−3^, and salt representative anion, Cl^−^, freeze-dried sediments were extracted in order to measure ions trapped within the pore and OM matrix. NH_4_^+^, NO_2_^−^, Cl^−^ and SO_4_^−2^ were extracted using DI water. PO_4_^−3^ was extracted from sediment using a solution of 0.01 M CaCl_2_ and 0.001 M DTPA (chelating agent) to enhance extraction across a varying range of sample OM and pH values. 5 g samples were extracted with 30 ml of extractant in acid washed 100 ml glass jars. Samples were subjected to 5 min sonication followed by 2 h of shaking on a lateral shaker before extracts were vacuum filtered sequentially, through 0.7 µm and 0.45 µm filter papers. Extracts were made up to 50 ml in volumetric flasks using deionised water.

NH_4_^+^ was determined colorimetrically in 0.45 µm filtered samples using the adapted Berthelot method as described by Cogan et al. (2014), and PO_4_^−3^ 0.22 µm filtered extracts were analysed by ICP-OES as described by Ivanov et al. (2010). Chloride and sulfate were determined using Ion chromatography (Dionex™ ICS 1500). The system had AG 22 guard column, an IonPac AS22 4*25 cm column, an ASRS300 4 mm suppressor column, a conductivity detector and utilised Chromeleon software for data acquisition and processing. Extracts were filtered through 0.22 μm syringe filters prior to pre-determined dilutions with DI water. The dilution factors were confirmed after preliminary concentration range testing using a representative sample from the salt marsh sediments (1 in 100 dilution required) and a sample from the intertidal sediments (1 in 10 dilution required). For IC analysis, an eluent was prepared using 4.5 mM sodium carbonate: 1.4 mM sodium bicarbonate dissolved in Milli-Q pure water with subsequent vacuum filtration through a 0.22 µm filter and eluent was pumped through the system at a flow rate of 1.2 ml/min, maintaining a temperature of 30 °C for a total run time of 15 min. An external calibration curve was generated with a mixed anion standard prepared for target analytes (Cl^−^, SO_4_^−2^), using sodium chloride and sodium sulfate salts. All standards and samples were injected and analysed in triplicate.

### Data processing and statistical analysis

All data processing and statistical analysis for testing of geochemical data were completed using XLSTAT software (https://www.xlstat.com). Descriptive statistics provided mean, standard deviations and ranges of data for each variable tested. Raw and log transformed data means (n = 3 analytical replicates) for variables were tested for normality using the Shapiro Wilks test. A spearman’s correlation matrix was generated using log transformed data to test the strength of pairwise relationships between measured soil variables. Significant changes in measured soil variables were explored between groups using the non-parametric Kruskal–Wallis test. Multiple pairwise comparisons using the Steel–Dwass-Critchlow-Fligner procedure ensured variables that differed significantly only between all groups were resolved and chosen as explanatory variables in ordination analysis. This approach reduces the number of environmental variables to measurements that differ most significantly across sites or gradients, thus selecting properties potentially having the highest impact on microbial communities and functionality.

Multicolinearity analysis was run on the selected variables to search for potential co-linearity where the variance inflation factor (VIF) exceeds a value of 10. Co-linearity can cause issues in ordination analysis where prior strong correlations (r > 0.700) between variables in a generated model may reduce accuracy due to redundancy in one or more of the variables (e.g. A pair of predictor variables with an r = 0.850 in the same model will inevitably express a strong influence for both as one increases) (Prunier et al. 2015; Alves et al. 2017). Geochemical variables for PCA were chosen after examining covariation results and representative variables were selected (pH, % OM, NO_2_^−^, PAH, TP, Clay, EC) for OM fractions, metals, inorganic particles, anthropogenic influences, nutrients and sediment chemistry, while minimizing inter-correlations where possible. In order to identify the geochemical properties that contribute most to the separation between zones, we applied Kruskal Wallis testing and (Kruskal and Wallis, 1952).

## Results

### Sediment geochemistry

**Fig. 3:**
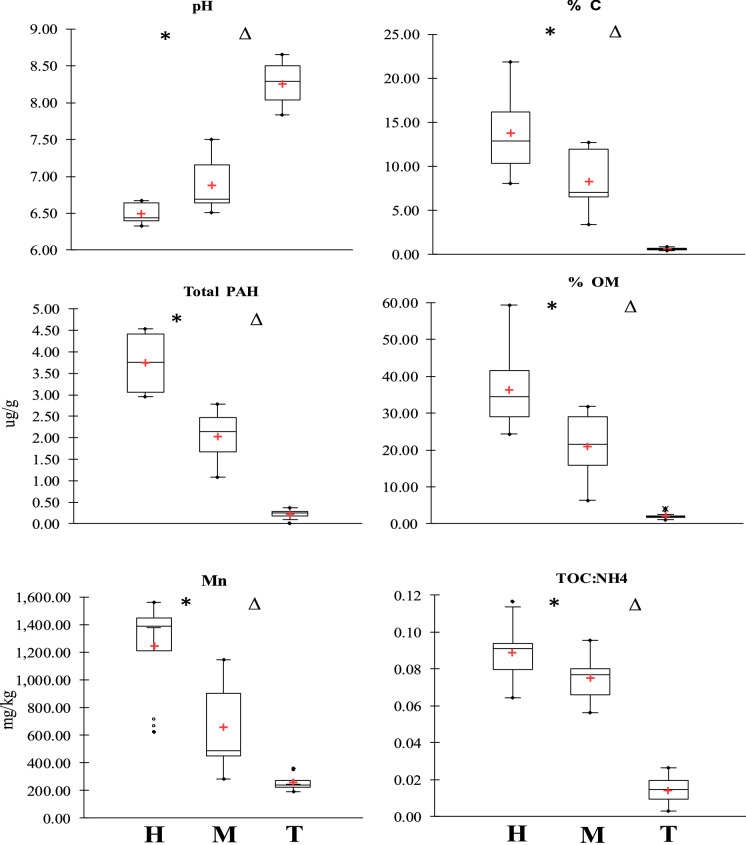
Environmental variables with significant changes (significant at level alpha = 0.05) between adjacent grouped sample zones H (n = 12), M (n = 15) and T (n = 12) (i.e. H vs M, M vs T and H Vs T). Significant differences between H and M, M and T zones is denoted by ‘*’ and ‘Δ’ respectively, above the relevant boxplot

All sample sites were assessed through analysis of 27 different physiochemical properties. Results for individual sample sites (means of a, b and c) are displayed in supplementary data Table S.1. Sample sites were grouped as discussed previously and results were tabulated accordingly as shown in Table 2. Considering the gradient from the Tidal mud zone (T), through the low-mid marsh (M) to the upper marsh (H), there were significant differences between all zones for many measured environmental variables as described by box plots in Fig. 3. % OM, % C and % H, all increased from zone T through to zone H, where %OM was significantly highest with a mean of 36.40 ± 10.03% (p < 0.05), reducing in zone M to 21.04 ± 8.51% and the lowest mean measured for zone T with 2.12 ± 0.19%. % C and % H followed similar trends as expected due to both being elemental constituents of OM and both having significantly high correlations with %OM (%C: r = 0.971, p < 0.001 and %H: r = 0.984, p < 0.001—see Table 3).

**Table 2:**
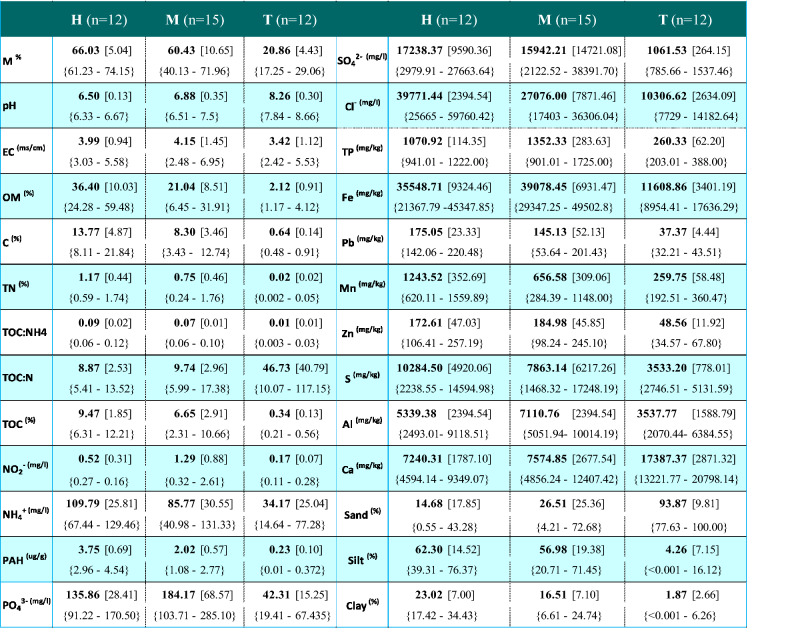
Table displaying the mean and standard deviation of measurements attained for abiotic variables for grouped sample sites representing gradient zones

For each variable the mean ‘**bold**’ is reported, followed by the standard deviation in square brackets ‘[SD]’ and the range underneath in curled brackets ‘{Range}’

*M %* Moisture, *pH* pH, *EC (ms/cm) *electrical conductivity, *OM (%) *organic matter, *C (%)*total Carbon, *TN (%)*, total Nitrogen, *TOC: NH4* total organic Carbon to Ammonia ratio, *TOC: N* total organic Carbon to total Nitrogen ratio, *TOC (%)* total organic Carbon, *NO2- (mg/l)* Nitrite, *NH4 + (mg/l) *Ammonia, *PAH (ug g) *poly-aromatic hydrocarbon, *PO43- (mg/l) *Phosphate, *SO42- (mg/l) *Sulfate, *Cl- (mg/l)* Chloride, *TP (mg/kg) *total Phosphorus, *Fe (mg/kg)* total Iron, *Pb (mg/kg) *total Lead, *Mn (mg/kg)* total Manganese, *Zn (mg/kg)* total Zinc, *S (mg/kg) *total Sulfur, *Al (mg/kg) *total Aluminium, *Ca (mg/kg) *total Calcium, *Sand (%) *total Sand, *Silt (%)* total Silt, *Clay (%) *total Clay

The pH decreased from the tidal zone to the upper marsh as % OM increased, where H had the lowest range of pH 6.33–6.67 when compared to M and T, pH 6.51–7.50 and 7.84–8.66 respectively. Table 3 displays Spearman’s correlation analysis results of PCA variables. Correlation results between all variables are displayed in Tables S2.a and S2.b. Bulk chemical parameters with significantly (p < 0.0001) strong positive correlations to % OM included %TOC, %N and %M. %TOC and %N had a significantly higher concentration in zone H (TOC = 9.47 ± 1.85% and N = 1.17 ± 0.44) than both M (TOC = 6.65 ± % and N = 0.75 ± 0.46%) and T (TOC = 0.34 ± 0.13% and N = 0.02 ± 0.02%). PAHs had a significant relationship with the presence of organic carbon (r = 0.811, p < 0.001), where concentrations were significantly highest in the upper marsh zone H, having a mean of 3.75 ± 0.69 ug/g and relatively lower in zone M (2.02 ± 0.57 ug/g) and zone T (0.23 ± 0.10 ug/g). Physical sediment properties were analysed using % sand, % silt and % clay contents. T zones had a significantly higher mean % sand content (T = 93.87 ± 9.81%) with a large decrease on the gradient approach towards the upper marsh zone and St. Anne’s golf club border, also showing higher variability (H = 14.68 ± 17.85%, M = 25.51 ± 25.36%). % Silt and % clay were strongly positively correlated and negatively correlated with % sand, thus both % silt (T = 4.26 ± 7.15%, M = 56.98 ± 19.38%, H = 62.30 ± 14.52%, where p < 0.001) and % clay were significantly higher in the H and M zones (T = 1.87 ± 2.66%, M = 16.51 ± 7.10%, H = 23.02 ± 7.00%).

**Table 3:** Spearman’s correlation results showing the relationships between sediment geochemical variables

	pH		EC ^(ms/cm)^		OM ^(%)^		N02^− (mg/l)^		PAH ^(ug/g)^		TP ^(mg/kg)^		Clay ^(%)^	
M ^(%)^	**− 0.8437**	< *0.0001*	− 0.0735	*0.6555*	**0.9136**	< *0.0001*	**0.7032**	< *0.0001*	**0.7806**	< *0.0001*	**0.8124**	< *0.0001*	**0.5894**	*0.0001*
pH	**1.0000**	*0.0000*	− 0.2013	*0.2183*	**− 0.9138**	< *0.0001*	**− 0.5777**	*0.0002*	**− 0.8722**	< *0.0001*	**− 0.6512**	< *0.0001*	**− 0.7323**	< *0.0001*
EC ^(ms/cm)^	− 0.2013	*0.2183*	**1.0000**	*0.0000*	0.0569	*0.7301*	− 0.0096	*0.9539*	0.2719	*0.0941*	0.0800	*0.6273*	**0.5402**	*0.0005*
OM ^(%)^	**− 0.9138**	< *0.0001*	0.0569	*0.7301*	**1.0000**	*0.0000*	**0.6650**	< *0.0001*	**0.8952**	< *0.0001*	**0.6981**	< *0.0001*	**0.6156**	< *0.0001*
C ^(%)^	**− 0.8995**	< *0.0001*	− 0.0193	*0.9073*	**0.9709**	< *0.0001*	**0.6968**	< *0.0001*	**0.8538**	< *0.0001*	**0.7181**	< *0.0001*	**0.5910**	*0.0001*
N ^(%)^	**− 0.8925**	< *0.0001*	0.0503	*0.7604*	**0.9431**	< *0.0001*	**0.6704**	< *0.0001*	**0.8037**	< *0.0001*	**0.7278**	< *0.0001*	**0.6339**	< *0.0001*
TOC:NH4	**− 0.8588**	< *0.0001*	0.1338	*0.4152*	**0.8330**	< *0.0001*	**0.6182**	< *0.0001*	**0.8327**	< *0.0001*	**0.6851**	< *0.0001*	**0.7170**	< *0.0001*
TOC:N	**0.7028**	< *0.0001*	− 0.1268	*0.4402*	**− 0.7484**	< *0.0001*	**− 0.5453**	*0.0004*	**− 0.6023**	< *0.0001*	**− 0.5324**	*0.0006*	**− 0.5267**	*0.0007*
TOC ^(%)^	**− 0.9025**	< *0.0001*	− 0.0168	*0.9192*	**0.9551**	< *0.0001*	**0.6992**	< *0.0001*	**0.8109**	< *0.0001*	**0.7818**	< *0.0001*	**0.6005**	< *0.0001*
N02^− (mg/l)^	**− 0.5777**	*0.0002*	− 0.0096	*0.9539*	**0.6650**	< *0.0001*	**1.0000**	*0.0000*	**0.5659**	*0.0002*	**0.7729**	< *0.0001*	**0.4408**	*0.0054*
NH4^+ (mg/l)^	**− 0.7616**	< *0.0001*	− 0.1125	*0.4940*	**0.8470**	< *0.0001*	**0.6824**	< *0.0001*	**0.7023**	< *0.0001*	**0.7446**	< *0.0001*	**0.4725**	*0.0027*
SO_4_^2− (mg/l)^	**− 0.7574**	< *0.0001*	0.1373	*0.4033*	**0.8494**	< *0.0001*	**0.7652**	< *0.0001*	**0.7758**	< *0.0001*	**0.8152**	< *0.0001*	**0.6284**	< *0.0001*
Cl^− (mg/l)^	**− 0.8568**	< *0.0001*	0.0215	*0.8968*	**0.9423**	< *0.0001*	**0.7957**	< *0.0001*	**0.8556**	< *0.0001*	**0.7192**	< *0.0001*	**0.5663**	*0.0002*
PAH ^(ug/g)^	**− 0.8722**	< *0.0001*	0.2719	*0.0941*	**0.8952**	< *0.0001*	**0.5659**	*0.0002*	**1.0000**	*0.0000*	**0.5889**	*0.0001*	**0.7624**	< *0.0001*
PO_4_^3− (mg/l)^	**− 0.6423**	< *0.0001*	0.2059	*0.2078*	**0.6893**	< *0.0001*	**0.7739**	< *0.0001*	**0.6103**	< *0.0001*	**0.7520**	< *0.0001*	**0.6302**	< *0.0001*
TP ^(mg/kg)^	**− 0.6512**	< *0.0001*	0.0800	*0.6273*	**0.6981**	< *0.0001*	**0.7729**	< *0.0001*	**0.5889**	*0.0001*	**1.0000**	*0.0000*	**0.6635**	< *0.0001*
Fe ^(mg/kg)^	**− 0.6923**	< *0.0001*	**0.3271**	*0.0426*	**0.5808**	*0.0001*	**0.4917**	*0.0017*	**0.6098**	< *0.0001*	**0.7628**	< *0.0001*	**0.8439**	< *0.0001*
Pb ^(mg/kg)^	**− 0.6917**	< *0.0001*	0.1446	*0.3786*	**0.7986**	< *0.0001*	**0.6571**	< *0.0001*	**0.7477**	< *0.0001*	**0.8346**	< *0.0001*	**0.6479**	< *0.0001*
Mn ^(mg/kg)^	**− 0.9220**	< *0.0001*	0.0754	*0.6471*	**0.9109**	< *0.0001*	**0.5672**	*0.0002*	**0.8584**	< *0.0001*	**0.5808**	*0.0001*	**0.6046**	< *0.0001*
Zn ^(mg/kg)^	**− 0.6486**	< *0.0001*	0.2248	*0.1683*	**0.6387**	< *0.0001*	**0.5447**	*0.0004*	**0.5975**	< *0.0001*	**0.8677**	< *0.0001*	**0.7386**	< *0.0001*
S ^(mg/kg)^	**− 0.4561**	*0.0038*	− 0.2694	*0.0973*	**0.6496**	< *0.0001*	**0.4818**	*0.0021*	**0.4934**	*0.0016*	**0.4410**	*0.0053*	0.1053	*0.5220*
Ca ^(mg/kg)^	**0.7050**	< *0.0001*	**− 0.3879**	*0.0152*	**− 0.6002**	< *0.0001*	**− 0.5020**	*0.0013*	**− 0.6653**	< *0.0001*	**− 0.7486**	< *0.0001*	**− 0.9091**	< *0.0001*
Al ^(mg/kg)^	− 0.3113	*0.0542*	**0.5060**	*0.0012*	0.1417	*0.3882*	0.2605	*0.1092*	0.1782	*0.2765*	**0.4527**	*0.0041*	**0.5675**	*0.0002*
Sand ^(%)^	**0.7847**	< *0.0001*	**− 0.3800**	*0.0176*	**− 0.6698**	< *0.0001*	**− 0.4037**	*0.0113*	**− 0.7624**	< *0.0001*	**− 0.6991**	< *0.0001*	**− 0.9451**	< *0.0001*
Silt ^(%)^	**− 0.7981**	< *0.0001*	0.2086	*0.2018*	**0.7362**	< *0.0001*	**0.4969**	*0.0015*	**0.6975**	< *0.0001*	**0.8023**	< *0.0001*	**0.8187**	< *0.0001*
Clay ^(%)^	**− 0.7323**	< *0.0001*	**0.5402**	*0.0005*	**0.6156**	< *0.0001*	**0.4408**	***0.0054***	**0.7624**	** < *0.0001***	**0.6635**	< *0.0001*	**1.0000**	*0.0000*

The analysis (n = 39) represents sample sites in all zones pooled for north and south lagoons. See Table 2 for abbreviations. The ‘r’ value and ‘p’ value (value in Italics) are displayed for each variable relationship (separated by broken line) i.e. pH vs M ^(%)^ → **[r]** = -0.8437 **[p] = ** < *0.0001*

Silt and clay fractions showed strong and significant (p < 0.0001) covariance with metals Fe (r = 0.863 and r = 0.844), Pb (r = 0.785 and r = 0.648) and Zn (r = 0.889 and r = 0.739) respectively. Silt displayed stronger relationships with OM, N, TP and NH4 + when compared to clay. However, clay particles were more strongly associated with the presence of PAH. Sediment nutrients including NO_2_^−^, NH_4_^+^ and SO_4_^2−^ were significantly (p < 0.0001) higher in zones H and M compared to T. NO_2_^−^ displayed significant differences between all zones where the highest mean was recorded in zone M (1.29 ± 0.88 mg/l, p < 0.001), with a strong contrast to lower concentrations across H and T (H = 0.52 ± 0.31 mg/l and T = 0.17 ± 0.07 mg/l). Available PO_4_^3−^ was highest in M zone at 184.17 ± `68.57 mg/l, significantly higher than zone T, but not H where the mean was 135.86 ± 28.41 mg/l. Following a similar trend to bulk chemical species, all nutrients had strong correlations to %OM (NO_2_^−^: r = 0.665, PO_4_^3−^: r = 0.689, SO_4_^2−^: r = 0.849, NH_4_^+^: r = 0.847, all p < 0.001), also evident with measured Cl^−^ (r = 0.942, p < 0.001), where concentration linearly increased from zones T up to H (T = 10,306 pm ± 2634.09 mg/l, M = 27,076 ± 7871.46 mg/l and H = 39,771 ± 12,421.40 mg/l). Variation between sites for all aforementioned anions was not reflected in the EC (ms/cm) measurements, with similar values recorded for all zones where H = 3.98 ± 0.94 ms/cm, M = 4.15 ± 1.45 ms/cm and T = 3.42 ± 1.12 ms/cm. Mn was significantly (p < 0.001) different across all zones, highest in H zone at 1,243.52 ± 352.69 mg/kg, decreasing by ~ 50% in zone M with increased variability at 656.58 ± 309.06 mg/kg and the lowest in zone T with a mean of 259.75 ± 58.48 mg/kg. Pb and S were highest in zone H zones (Pb = 174.05 ± 23.33 mg/kg, S = 16,449.77 ± 4920.06 mg/kg), mean Al, Zn, Fe and total P (TP) were highest in M zones, with both Al (p < 0.03) and TP (p < 0.05) having significantly highest concentrations in the M zone (Al = 7,110.76 ± 2394.54 mg/kg, Zn = 184.98 ± 45.85 mg/kg, Fe = 39,078.45 ± 6931.47 mg/kg and P = 1352.33 ± 283.63 mg/kg). Ca was significantly highest in the tidal zone T at 17,387.37 ± 2871.32 mg/kg in contrast to zones H (7,240.31 ± 1,787.10 mg/kg) and M (7,574.85 ± 2677.54 mg/kg). All other metals excluding Ca and Al displayed significant, strong and positive relationships with carbon content. Ca was strongly and negatively correlated with carbon (e.g. %TOC and Ca: r = -0.643, p < 0.001) while Al did not display any statistical relationship with carbon.

### PAH distributions

**Fig. 4:**
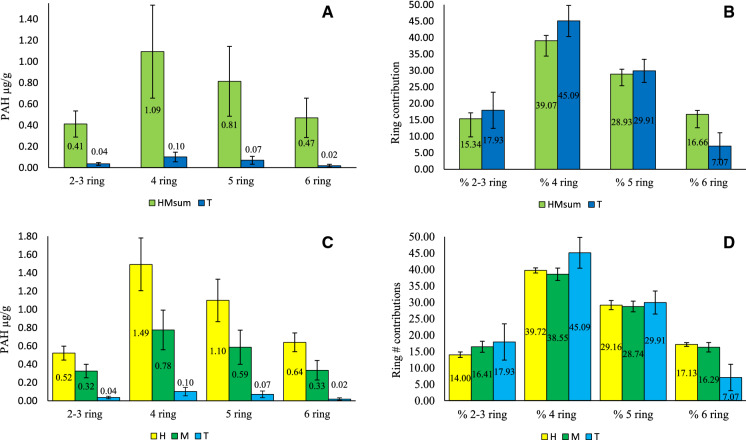
Graphs displaying results for distribution and concentration of PAH by ring number categories and contribution of ring category by % mass of total PAH in respective sample zones: **A** Concentration of PAH µg/g by ring category in vegetated salt marsh sediments, defined by the sum of zones H and M [HM_sum_], and in the intertidal zone T sediments. **B** % contribution of ring category to total PAH concentration in HM_sum_ and zone T sediments. **C** Concentrations of PAH by ring categories in sediments of zones H, M and T. **D** % contribution of ring category to total PAH concentration in sediments of zones H, M and T

Total PAH (µg/g) concentrations and all ring number (µg/g) concentrations were significantly (p < 0.0001) higher in HM_sum_ than zone T sediments (Figs. 3 and 4). Indeed, 2–3 ring (HM_sum_ = 0.41 ± 0.12 µg/g, T = 0.04 ± 0.01 µg/g), 4 ring (HM_sum_ = 1.09 ± 0.44 µg/g, T = 0.10 ± 0.05 µg/g), and 5 ring (HM_sum_ = 0.81 ± 0.33 µg/g, T = 0.07 ± 0.04 µg/g) were over 10 times higher, 6 ring (HMsum = 0.47 ± 0.19 µg/g, T = 0.02 0.01 µg/g) over 20 times higher in HM_sum_ sediments. Ring contributions of % 2–3 ring (HM_sum_ = 15.34 ± 1.82%, and T = 17.93 ± 5.52%), % 4 ring (HM_sum_ = 39.07 ± 1.58%, and T = 45.09 ± 4.70%, significant at p < 0.001) and % 5 ring (HM_sum_ = 28.93 ± 1.51%, and T = 29.91 ± 3.51%) were all highest in zone T sediments, albeit higher variability. % 6 ring PAHs contributed (HM_sum_ = 16.66 ± 1.19%, and T = 7.07 ± 4.03%) significantly (p < 0.0001) higher in HM_sum_ sediments than zone T sediments.

Inter-zone comparison on the elevation gradient between zones H, M and T showed total PAH and all ring number concentrations to be significantly (p < 0.0001) highest for zone H (2–3 ring = 0.52 ± 0.08 µg/g, 4 ring = 1.49 ± 0.29 µg/g, 5 ring = 1.10 ± 0.23 µg/g, and 6 ring = 0.64 ± 0.10 µg/g), with zone M (2–3 ring = 0.32 ± 0.07 µg/g, 4 ring = 0.78 ± 0.02 µg/g, 5 ring = 0.59 ± 0.19 µg/g, and 6 ring = 0.33 ± 0.12 µg/g) significantly (p < 0.0001) higher in all ring number classes than zone T (2–3 ring = 0.04 ± 0.01 µg/g, 4 ring = 0.10 ± 0.05 µg/g, 5 ring = 0.07 ± 0.04 µg/g, and 6 ring = 0.02 ± 0.02 µg/g). Highest % contribution of PAH ring category to all zones was 4 ring (H = 39.72 ± 0.76%, M = 38.55 ± 1.88%, and T = 45.09 ± 4.70%.) which was significantly different between zones with decreasing contribution from zone T (p = 0.024) → zone H (p = 0.032) → zone M. % contribution of 5 ring PAH followed the same order of contributions (H = 29.16 ± 1.37%, M = 28.7 ± 1.63%, and T = 29.91 ± 3.5%), albeit there was no statistical significance between zones. In zone T sediments, % 2–3 ring contribution to total PAH was significantly higher than zone H (p = 0.012) and higher than zone M at 17.93 ± 5.52%, while 6 ring PAH in zone T had the lowest contributions at 7.07 ± 4.03%. In zone M, % 2–3 ring (16.41 ± 1.68%, p < 0.0001) contribution was also significantly higher than in zone H and 6 ring PAH concentrations in sediments was 16.29 ± 1.43%, over twice the content in zone T (p < 0.0001). Zone H sediments had the highest (p < 0.0001) % 6 ring contribution in sediments with 17.13 ± 0.55%, while 2–3 ring content was 14.00 ± 0.84%.

Effects range low (ErL) and effects range median (ErM) concentrations are used as numerical predictor values for toxicity in marine sediments. ErL and ErM values for low molecular weight (LMW) PAHs (2–3 ring) are 0.55 µg/g and 3.16 µg/g respectively, while ErL and ErM for high molecular weight (HMW) PAHs (4–6 ring) are 1.70 µg/g and 9.60 µg/g respectively. ErL and ErM values for total PAH (16 priority PAHs) are 4.02 µg/g and 44.79 µg/g respectively (Long et al. 1998; Neff et al. 2011). Mean values for HMsum sediments were above the ErL for 2–3 ring PAH at 0.85 µg/g and below the ErM. Zone T values for LMW PAH were below both ErL and ERM criteria. HMW ErL in HMsum sediments was surpassed with a value of 4.93 µg/g while remaining below the ErM. Individual zones H, M and T were below the ErL and ErM criteria for both LMW and HMW PAH. The total PAH for HMsum sediments was in the ErL and ErM range with a value of 5.77 µg/g. The defined zones H, M and T had total PAH values below the ErL and ErM range, while sample sites SM2 and SM8 in zone H were in the ErL-ErM range with values for total PAH of 4.43 µg/g and 4.17 µg/g respectively. In this study, comparisons showed no statistically significant difference in concentrations of total PAH µg/g and mean ring groups between NL (4.49 ± 1.87 µg/g PAH) and SL (4.54 ± 2.06 µg/g PAH). However, the contributions of % 4 ring PAH (NL = 39.05, SL = 41.76, p = 0.025) was significantly higher in SL than in NL sediments.

### Principal component analysis (PCA) and elevation results

**Fig. 5:**
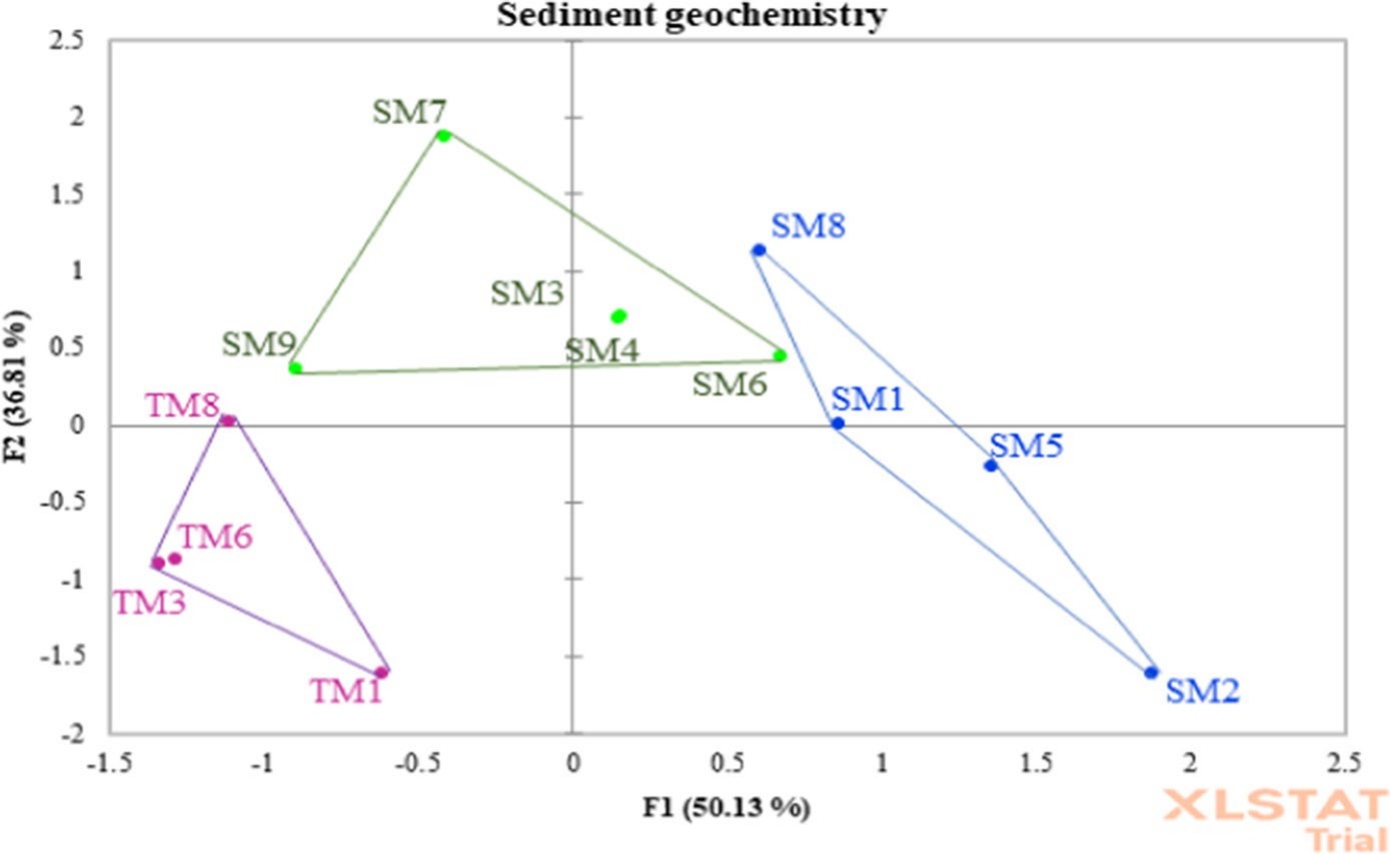
PCA of geochemical variables. Points are coloured for respective group zones and the convex hulls are drawn and highlighted for each of the groupings. The sample sites showed separation into groups representing upper marsh (H), mid-lower marsh (M) and tidal flats (T) zones, revealing similar patterns between samples in multivariate space. There was a clear and consistent separation of sample sites along the first principal component axis (F1) with 86.94% variance explained for combined axis (F1 and F2)

Principal component analysis by spearman’s correlation matrix was carried out using selected environmental predictor variables pH, % OM, NO_2_^−^ mg/l, PAH µg/g, TP, % Mud (Sum of Clay and Silt), EC ms/cm. Plotted sample sites and respective zones showed a distinct separation along the first axis (F1) with no direct overlaps in multivariate space (Fig. 5). The predictor variables PCA explained 86.94% variance between axis F1 (50.13%) and F2 (36.81%) and provided evidence of more separation between all groups. i.e. the influence of one or more variables has a more profound significance on a group through quantitative measurements or presence, with respect to other groups. The primary reason for such separation of groups in ordination is due to removal of multiple co-linear variables from the original data set. Using multicolinearity analysis, predictor variables were tested using the VIF and subsequent variables were selected to achieve VIF < 10 for all variables, while maintaining a relationship to the model. The remaining variables pH, % OM, NO_2_^−^ (mg/l), PAH (µg/g), TP (mg/kg), % Mud, and EC ms/cm each have a relationship on a scale of positive or negative with individual sites in respective groups, without displaying redundancy through excessive co-linearity with another predictor variable. All variables were tested for significant differences between groups, thus all chosen variables had a significantly highest or lowest value within one of the 3 group zones.

**Fig. 6:**
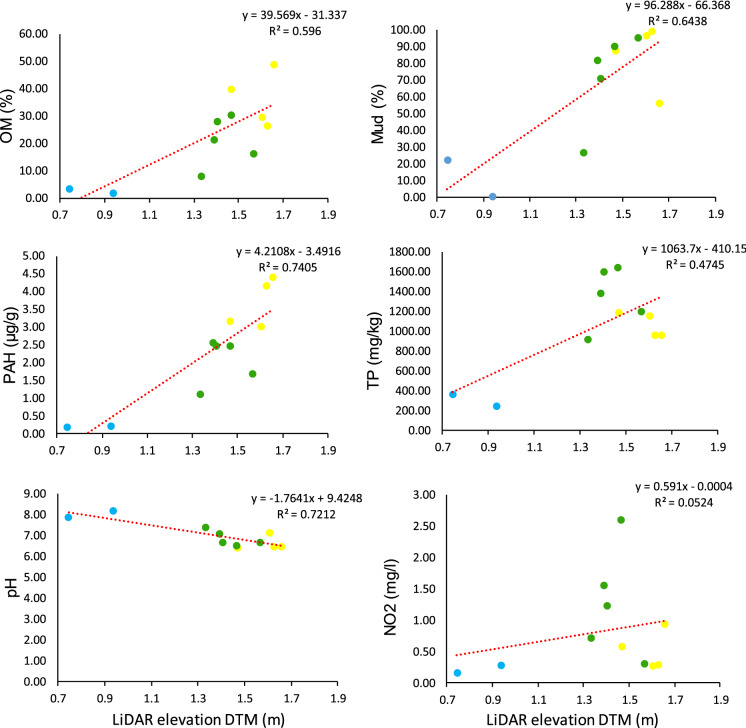
Scatter plots with corresponding trend lines of PCA predictor variables (y-axis) versus LiDAR elevation DTM (m) measurements. Coloured points correspond to transect zones for each site: Yellow = zone H (upper marsh); Green = zone M (mid-low marsh); Blue = zone T (intertidal mudflats). Two zone T samples have been omitted as measurements could not be accurately assigned outside of the LiDAR scan where both sites were detected to exist < 0.7400 DTM (m)

The important result is validation of the predictor variables by confirmation of group clusters through arrangement of individual site co-ordinates in PCA ordination. The variables show a clear separation between H, M and T zones, thus geochemical gradients have evolved across the study site as a function of salt marsh accretion. Comparison within group ordination shows a greater distance between individual sites in groups M and H with respect to group T. This is a reflection of in group variation between site values for pH, % OM, NO_2_^−^ mg/l, PAH µg/g, TP, % Mud, and EC ms/cm, indicating heterogeneity in sediment chemistry. This is likely consequence of many factors affecting geochemistry including vegetation presence, perturbations in tidal inundation and lateral drainage of water from upper marsh zones due to the elevation gradients. Indeed, plots from Fig. 6 show strong positive linear relationship between site elevations and mass of present OM, PAH, TP and mud. Tighter clusters in-group T are indicative of less variation across sites with respect to the predictor variables. Abiotic results show significantly (where, p < 0.001 for each) higher pH, and lower % OM, PAH (ug/g), TP, and NO_2_^−^ in zone T compared to M and H, thus reflected in the groupings and separation on axis F1. Nitrite is highest in zone M in comparison to zones H and T, explained by separation vertically on axis F2. Mean EC did not display any significant difference between zones; however, EC was highest in zone M (4.14 ms/cm) when compared to zones H (3.99 ms/cm) and T (3.42 ms/cm).

NL sample sites SM7, SM8, SM9 and TM 8 consistently have the highest F2 and lowest F1, subsequently falling into multivariate space inhabited by all zone M sites. These NL sites display within group distance (SM8 – zone H, SM7 & SM9- zone M and TM8-zone T) from other group representative sites from the SL sediments. The occurrence of this ordination indicates that differences between the lagoons is driven by other hydrological factors in addition to elevation. NL sample sites (n = 12) were grouped and tested against SL samples sites (n = 27) to explore differences in sediment properties between lagoons subject to different hydrological regimes (Table S.3). In the NL sediments, Al (8144.53 mg/kg) content was nearly 2 times higher than the SL (Al = 4276.03 mg/kg), while EC (4.97 ms/cm) was significantly higher than in the SL (Al = 3.39 ms/cm). Total S was over 4 times higher (p = 0.002) in the SL (9513.31 mg/kg) than the NL (2241.67 mg/kg) subsequently reflected in sulfate content 7 times higher for SL than NL sediments with values of 15,979.08 mg/kg and 2274.73 mg/kg respectively. Indeed, values for % OM, % C, % N, % TOC, NO_2_^−^, Cl^−^, TP, Pb, Mn, and Zn, were significantly higher in the SL than the NL, which aligns with results for many variables in a previous study of BI’s lagoon sediments by Grey et al. (2021). Contrary to results in previous work, in this study the NL had higher clay (18.01%) and silt (43.226%) content, and lower sand (38.76%) when compared to the SL (% clay = 12.23, % silt = 42.02, % sand = 45.75).

In summary, sediment geochemistry showed a sharp and expected change with transition from intertidal to vegetated supratidal sediments where elevation increased. There were distinct and significant increasing concentrations of % OM, % C, % TOC, %N, Mn, and PAH on the upward elevation gradient (from zone T through zones M and H). C content was 16 fold higher overall in vegetated (3.43 -21.84%) than uninhabited (0.21–0.56%) sediments, while TN was over 50 times higher (0.24–1.76%), more specifically increasing in % mass on approach to the upper salt marsh with distance from the tidal flats sediments zone T (0.002–0.05%). TOC:N ratio which represents bulk organic carbon and N was 5 times lower (p < 0.05) in un-vegetated zone T relative to vegetated zones. However, TOC: NH_4_^+^ ratio, was significantly (p < 0.050) different between zones with a highest mean in upper marsh zone H (0.09), decreasing on approach (Zone M = 0.07) to the tidal flats (0.01). The retention of water, metals, PAHs, chloride ions, NH_4_^+^, PO_4_^3−^ and SO_4_^2−^ increased with elevated C concentrations, concurrently where pH significantly decreased, with all variables showing strong covariance (Table 3). Total PAH concentrations were high in all SM samples. Similarly, metals, sediment nutrients- NH_4_^+^, PO_4_^3−^, SO_4_^2−^ and Cl^−^ ion concentrations are tightly linked to %C distributions as accumulation increases linearly, except NO_2_^−^ which was highest in zone M. % Mud (sum of % clay and silt) content also increased with elevation despite receiving less tidal inundation suggesting particle entrapment is enhanced by higher elevation and vegetated sediments. Figure 6 displays scatter plot graphs describing the linear relationship between selected variables and elevation measurements for 11 sample sites within the bounds of the LiDAR scan data (excluding 2 south lagoon intertidal samples TM3 and TM6 as out of bounds). Elevation has the strongest positive relationships with PAH µg/g (R^2^ = 0.7405), % Mud (R^2^ = 0.6438) and % OM (R^2^ = 0.5960), while TP (R^2^ = 0.4745) was weakly related to elevation over all the sites. The transition from zone T to SM sediments showed a high increase in TP, where the highest TP was determined for zone M, and reflected in the weaker R^2^ value for the elevation vs TP plot (Fig. 6). NO_2_^−^ mg/l and EC ms/cm displayed no linear trend with the current data set. Conversely, pH (R^2^ = 0.7405) displayed a strong negative relationship with elevation, where pH decreases towards vegetated and high OM sediments showing a relatively low slope indicating a slower rate of change.

## Discussion

### Bulk OM, metal, PAH distributions and influence of elevation

In the context of biogeochemical cycling in tidal marsh environments, the distributions of bulk sediment properties such as OM, C, N, fluvial particles, and total metals can represent fractions contributing to longer-term deposition of autochthonous, allochthonous and anthropogenic inputs to accreting sediment. Indeed, in the lagoon sediments of Bull Island, these components increase in mass contribution as elevation increases, in addition to the increase in vegetation cover. This is in contrast to abiotic transient parameters in sediments such as nutrients and oxygen availability, which are subject to regular fluctuations especially in dynamic coastal systems under hydrological and climatic change, but also due to constraints from biotic demands. In essence, the accumulation of OM and metals arises in sediments from initial introduction, chemical transformations and eventual immobilisation through biotic uptake processes and abiotic complexes, albeit with great spatial heterogeneity even at localised scales (Seyfferth et al. 2020). Accumulated OM and metabolically important metals provide a sink of substrate for both vegetation and microbial assimilation when redox conditions permit. These same redox conditions can increase solubility and liberation of both pollutant class metals, and organic contaminants.

In this current study, relationships between OM and total bulk metals were significantly and positively high including Fe and Zn. However, stronger relationships existed with OM and Pb, Mn, S and total P, while the distributions of Fe and Zn were more strongly associated with each other and the presence of clay. Clay and OM also displayed a significantly strong relationship. In solid form, Fe is an element in mineral clays and also exists in sediments as Fe-oxides in many forms (e.g. Goethite, Hematite and ferrihydrite) with both inorganic structures known to have a strong affinity for OM (Adhikari and Yang 2015) and other metals under redox dependant conditions, especially at pH > 5 (Rieuwerts et al. 1998). Conversely, Fe-oxides can catalyse the transformation and decomposition of OM to free radicals under Fenton oxidation like conditions where Fe-oxides readily form on the roots of SM vegetation (Yu and Kuzyakov 2021). The formation of Fe-oxide and metal complexes may explain strong relationships with Fe and other metals (Sundby et al. 2003; Waychunas et al. 2005). Relationships between OM and concentrations of metals in Bull island’s sediments have been previously reported (Williams et al. 1994; Doyle and Otte 1997; Grey et al. 2021). Doyle and Otte (1997) reported accumulation of Fe, Zn and As around the rhizosphere of marsh vegetation, and adjacent to the burrows of lugworms. These biotic conduits facilitate metal precipitation through fluctuating redox conditions where oxygen exchange occurs in the sediment matrix. This same process has been documented in equally polluted Dutch marshes with elevated Zn concentrations (Otte et al. 1993; Jacob and Otte 2003). Another component of metal in clays is Al, which also forms Fe-Al oxide complexes in marine based sediments. In this study, we found Al to be most strongly associated with Fe distributions, but also clay and EC. Similarly, Al was highest in zone M where Fe, Zn, TP and EC were elevated which is further evidence of the differing geochemical conditions between the vegetated H and M zone sediments on the elevation gradient.

Zone M represents the mid-lower saltmarsh where the high tide mark is more frequent than in the more elevated zone H. The higher EC which represents sediment pore conductivity can be linked to higher seawater loading and subsequent ion dissolution, circumstances which invariably alters sediment geochemistry (Weston et al. 2010; Weston et al. 2011). These processes can promote intense biogeochemical cycling at the exchangeable rhizospheric sediment–water inter-face, which promotes fluctuating changes in redox. Similar constraints apply for metals such as Fe, Zn and TP which have been shown in other studies to mobilize in sediment pore water more readily than Pb and Mn under fluctuating redox conditions (Wiese et al. 1997; Gutiérrez et al. 2019; Kouassi et al. 2019). Constrained by climatic conditions and elevation, these dynamic conditions exist especially on the lower reaches of a salt marsh, in sediments receiving regular tidal inundation, and coupled to the presence of diverse vegetation. Seawater inundation provides a conduit network for biogeochemical cycling of C and N, and been shown in to enhance the dissolution of OM constituents and increase DOM concentrations in coastal sediment pore-water through increasing pH > 8 (Anderson et al. 2018). Recent evidence provided by Mueller et al. (2020) demonstrates how regularly flooded sediments with high mineral content act as a sink for active enzymes, which provides an enhanced decomposition matrix for OM turnover even under low oxygen availability (Mueller et al. 2020).

Highest C and TN content occurred in Zone H, where accumulation of Pb and PAH is especially high, evidently coinciding with higher fluvial deposits of clay and silt. Despite the increased elevation of zone H relative to the zone T, significantly higher contributions of clay, silt and PAH content to zone H suggest a considerable input from adjacent river and run-off sources. Fluvial sediment loadings to estuaries have been previously reported at higher levels during flooding events which additionally, would increase water volume into the spatially confined lagoons, thus extending high sediment loaded waters to upper marsh zones (Negrin et al. 2011; Alber and O’Connell 2019). These processes could explain higher PAH and metal concentrations on elevated sediments. Atmospheric deposition of PAHs is another contributor, arising from high levels of traffic in Dublin City and historically, through power generating stations burning fossil fuels at Poolbeg.

Sediments were categorized according to PAH contamination where all 9 salt marsh samples were placed in the high polluted category (i.e. 1ug -5 ug/g). Zone H was classified as highly polluted with all group sample sites exceeding concentrations greater than 3 ug/g and a pooled mean of 3.75 ug/g. Zone M mean PAH was 2.02 ug/g classified as high pollution and zone T mean was 0.223 ug/g categorised as moderately polluted. Sample site SM2 had the highest value PAH content at 4.43 ug/g and TM3 the lowest with 0.080 ug/g. The total PAH exceeds ErL in all zones investigated (i.e. HMsum, zones H, M and T), however, the effect linearly increases in elevating vegetated sediments where % OM, %C, TN, clay, silt and Pb contribute highest content. The surface features of the SM zones clearly enhance the deposition and retention of PAHs. Previous work has shown that most PAHs in Bull island and Dublin bay sediments derive from the combustion of biomass and fossil fuels, with deposition regimes largely driven by hydrological constraints influenced by mixing of marine and freshwater where the R. Liffey enters Dublin Bay (Grey et al 2021; Murphy et al. 2016). The distributions of PAH appear to suggest source and transportation dynamics which intertwine with the movement of fine fluvial particles, and likely with allochthonous OM.

OM exerts strong control over retention of PAH in these sediments, a property that clearly influences the accumulation of metals, especially Pb and Mn. As an element in sediments under fluctuating pH and redox, Mn can be readily immobilised as Mn-oxide crusts at the interface of O_2_ and water on the roots of vegetation, precipitated by microbial/algae mediated oxidation and active uptake by plants. These Mn-oxide particles provide additional adsorption sites for other metals and PAHs. The % contribution of ring structure of PAH to distribution in zones appears to follow a pattern where elevated and OM rich sediments have higher relative contributions from HMW PAH, especially evident when comparing zones HM_sum_ and T. Furthermore, at a more resolved level, we see highest contributions of % 4, 5 and 6 ring PAH in zone H compared to zone M, where 2–3 ring contributions are slightly higher. LMW PAH are more susceptible to dissolution, abiotic and biotic degradation (Ukalska-Jaruga et al. 2019). Sediment properties in the older and elevated sediments of zone H appear to enhance removal of LMW PAH and efficient immobilisation of HMW species (Chen et al. 2021). These processes are likely controlled by a combination of physiochemical and microbial interactions in sediment microcosms. Zone T displayed highest percentage 2–3 ring contributions while unexpectedly these sediments had highest percentage contributions of four and five ring, contrasting strongly with a low percentage 6-ring content. Intertidal sediment surfaces may have a strong role in OM, metal, particle and PAH during the spring/summer months when expansive algae blooms cover Bull Island’s lagoons. During periods of elevated sediment loading and lower water volumes, retention of dissolved and suspended materials could accelerate in zone T due to adsorption, and entrapment in algal biomass. Indeed, algae are key to C immobilisation on a global scale, while species have been shown to actively process PAH (Duan et al. 2015), uptake metals (Jitar et al. 2015), and precipitate Fe/Mn oxides on the surface of cells (Robbins and Corley 2005). Organic and inorganic constituents entrapped in the buoyant and microbially active algae necromass deposit onto marsh sediments with incoming high tides. Inevitably, this provides further bioavailable substrate, persistent anthropogenic inputs, and foreign microbiology into heterogeneous surface sediments (Mestre et al. 2018). In fact, key questions remain around the level of significance that algae biomass contributes to blue carbon stocks globally, both as contributor to sedimentary C stocks (Macreadie et al. 2017), and as a catalyst for microbial C mineralisation of seagrass detritus (Liu et al. 2020). Considering the pollution index assigned to zones in this study, there is value in studying the impact that such anthropogenically influenced geochemical constraints have on the structure and functioning of microbial communities.

Contrary to a previous study by Grey et al. (2021), both clay and silt were slightly higher in the NL sediments, while PAH did not show any significant difference in concentrations between lagoons. This may reflect the heterogeneity of the lagoon sediments with respect to deposition regimes of allochthonous sources of fluvial particles, which are predominantly dependant on supply from riverine transport. Particle deposition dynamics on sediments are constrained by tidal cycles, water sediment loading, water energy, and ability of the sediment surface to facilitate retention of particles i.e. elevation, OM content and presence of vegetation. This type of heterogeneity may be less profound with OM, nutrients and metals as these properties can have large autochthonous inputs through vegetation biomass, nutrient and mineral recycling.

Sample size and spatial distribution of sample locations must also be considered when comparing lagoons, as intertidal samples with greater distance from vegetated marshes, and positioned in higher water energy zones will have less potential for facilitating deposition. In this study, zone T samples were consistently taken on a lateral transect that incorporated intertidal sediments with a relatively close proximity to the salt marsh boundary. The previous study by Grey et al. (2021) had a wider, and more random spatial representation of the intertidal lagoon sediments. Additionally, in the NL there is clear visual evidence of lateral marsh expansion where, emergent Salicornia flats extend towards the natural lower elevated lagoon channel (Figure S.2a and d). This change in topographic features and difference in lagoon sizes (Grey et al 2021) will inevitably alter future deposition dynamics and geochemical signatures between lagoon sediments.

### Ion distributions in sediment

NH_4_^+^, PO_4_^3−^, SO_4_^2−^ and Cl^−^ ion concentrations are tightly linked to % C distributions as accumulation increases linearly from T → M → H. The stronger correlations between water soluble SO_4_^2−^ and bulk Fe, P, N and % OM would suggest S as an anionic species is significant across the study site and not limited in availability. OM degradation liberates the release of nutrients into bulk soil and inevitable transformations into ionic species across varying redox conditions. Thermodynamic constraints under long-term O_2_ limitations favours microbial C degradation coupled to SO_4_^2−^,Fe (III) and Mn (IV) reduction, reported to be the primary mode of metabolism in water logged anaerobic sediments found in marine wetlands and terrestrial peatlands (Lovley et al. 1993; Lamers et al. 2012; Pester et al. 2012; Antler et al. 2019). However, ROL in rhizosphere horizons of VCEs is a primary route of O_2_ introduction to sediments, inevitably altering redox states at the root-sediment interface at rates dictated by diurnal and seasonal cycles (Lai et al. 2012; Zhao et al. 2022). Therefore, SO_4_^2−^, Fe Mn and other metal concentrations may build up intermittently at the sediment-root interface in the context of high marsh autotrophy where active root transport liberates thermodynamically favourable electron acceptor O_2_. Additionally, abiotic oxidation of reduced species (e.g. H_2_S, FeS, and NH_4_^+^) also occurs during these periods, invariably, increasing localised concentrations of microbial and plant available nutrients such as SO_4_^2−^ and NO_2_^−^. Importantly, under similar anoxic/anaerobic sediment conditions, the presence of high concentrations of soluble Fe (III), Fe-oxides and organic –bound Fe(III), has been shown to inhibit methanogenesis and act as an important regulating factor in microbial OM decomposition, thus potentially reducing CH_4_ emissions (Herndon et al. 2015).

NH_4_^+^ increased linearly with carbon, it’s prominent sources as a by-product of microbial organic matter mineralisation, NO_2_^−^ reduction and nitrogen fixation, additionally providing a precursor for NO_2_^−^ in environments where ammonia oxidation can occur (Kowalchuk and Stephen 2001; Francis et al. 2007). Many salt marsh vegetation species have adapted to utilise NH_4_^+^ as a primary N source where its presence in periodically anoxic rhizospheres is the most abundant of available N species (Wang et al. 2014). All water-soluble nutrients (NH_4_^+^, PO_4_^3−^, SO_4_^2^ and NO_2_^−)^ were collinear (r > 0.700, p < 0.001), with NO_2_^−^ especially showing a significant increase in zone M. Furthermore, Cl- increased strongly with % C content in sediments, clearly highest in zone H which is in agreement with a recent study demonstrating the ability of natural OM in marine sediments to act as a sink for chlorine (Leri et al. 2015).

Considering higher elevation of zone H, less regularity in seawater supply and higher exposure to freshwater from precipitation, would thus suggest that accumulation of sediment ions is a combination of both retention and active uptake, most likely through plant and microbial biomass. Halophyte plants have a high capacity for ion, nutrient and metals storage as part of osmoregulation processes from root to shoot and adaptation to fluctuating nutrient supply. The elevated Cl^−^ in conjunction with OM is likely due to annual turnover of plant detritus. Similarly, microbes present in highly saline environments must upregulate osmotic responses within the cell by the accumulation of cytoplasmic ionic solutes to maintain integrity when accessing C and nutrient substrates from external sources. These are important biotic aspects in the context of carbon sequestration in such geochemically dynamic and anthropogenically impacted sediments. The redox dynamics associated with biogeochemical cycling in the rhizosphere horizon of sediment enhances C, nutrient, pollutant and metal accumulation around plant roots, which is the zone that accounts for the majority of plant–microbe interactions (Spivak et al. 2019). It is here that organic acids are released into sediments as root exudates by plants, in response to immediate sediment conditions including high salinity, anoxia and exchangeable metal (Pezeshki and DeLaune 2012; Adeleke et al. 2017). This process has been shown to decrease sediment pH, subsequently complexing with metals and increasing solubility for uptake but also for detoxification of metals such as Al and Pb (Ma 2000; Chen et al. 2003; Menezes-Blackburn et al. 2016; Magdziak et al. 2017). Importantly, under certain conditions such mechanisms enhance stabilisation of C in soils through physical protection against microbial degradation (Pronk et al. 2013; Feng et al. 2014). The upside for carbon storage in blue carbon sediments is the promotion of longer term accumulation of organo-metallic compounds through integration into living and dead plant biomass and facilitating the immobilisation of metals with OM and clay (David and Jones 1998; Pezeshki and DeLaune 2012). From the perspective of carbon sequestration, temporal redox changes are part of the process for long-term C burial and accretion. Thereafter, conditions for microbial C degradation are controlled by electron donor/acceptor species and selective C substrate mineralisation (Loomis and Craft 2010; Oni et al. 2015; Kelleway et al. 2016; Boye et al. 2017; Stumpner et al. 2018).

## Conclusions

Historically, nutrient, carbon, particulates and organic/inorganic pollutant inputs to Dublin Bay have been elevated due to riverine transport, industrial activity, sewage effluent and run-off associated with geographical proximity to a highly urbanised coastal region (Brennan 1991; Jeffrey et al. 1995; Murphy et al*.*, 2016b). Invariably, algae blooms are a common occurrence in the Tolka estuary and on the mud flats of Bull Island’s lagoons. This biomass is a prominent source of proteinaceous material, inevitably contributing to C, N, S and P sediment concentrations, especially during seasonal die-off when tidal surges facilitate the distribution of necromass to elevated sediments, and through outwelling. Questions remains as to the degree of influence that algae may exert in immobilising heavy metal, fluvial sediments and PAH from the water column. The accretion of sediment (elevation) and presence of vegetation in zones H and M facilitate more efficient trapping of particulate OM, silts and clays, all contributing as vehicles for C substrate and nutrients to support seasonal autotrophy. Indeed, organic root exudates from halophytes can provide additional C for substrate for biogeochemical cycling. Additionally, detoxifying metals such as Al and Pb (Ma 2000; Chen et al. 2003; Menezes-Blackburn et al. 2016; Magdziak et al. 2017), and importantly under certain conditions, increased stabilisation of C in soils through physical protection against microbial degradation (Pronk et al. 2013; Feng et al. 2014). Furthermore, the combination of these edaphic factors facilitates accumulation of PAHs, with evidence of long-term retention of HMW constituents in elevated sediment zones. The variation in OM and metal distribution from zone T to SM zones arise due to different hydrological regimes, spatial elevation, higher deposition rates, succession of vegetation and high burial rates. However, variations between different metals in the comparisons between marsh zones H and M is difficult to attribute to all of the factors mentioned above. Biotic factors such as vegetation composition, algae necromass, and sediment microbiology may play a larger role in metal distributions throughout the elevation gradients by catalysing sediment redox dynamics during C cycling.

## Supplementary Information

Below is the link to the electronic supplementary material.

Supplementary file1 (DOCX 1336 kb)

## Data Availability

All data generated or analysed during this study are included in this published article [and its supplementary information files]. Any other information required is available from the corresponding author on reasonable request.
