# Supplementary material for: Geochemical properties of blue carbon sediments through an elevation gradient: study of an anthropogenically impacted coastal lagoon
Source: Biogeochemistry. 2023 Feb 8;162(3):381–408. doi: 10.1007/s10533-022-00974-0 (PMC9971090; doi:10.1007/s10533-022-00974-0)
Supplement: Supplementary file 1 — Supplementary file1 (DOCX 1336 kb) [file 10533_2022_974_MOESM1_ESM.docx]

Supplementary Information

Geochemical properties of blue carbon sediments through an elevation gradient: Study of an anthropogenically impacted saltmarsh. (2022)

**Authors**: Anthony Grey^†^, Ricardo Costeira^‡^, Emmaline Lorenzo^§^**,** Sean O’Kane**^ǂ^**, Margaret V. McCaul**^ϕ^,** Tim McCarthy**^ǂ^,** Sean F. Jordan ^ϕ^**,** Christopher C. R. Allen^‡^, Brian P. Kelleher^†*^.

| 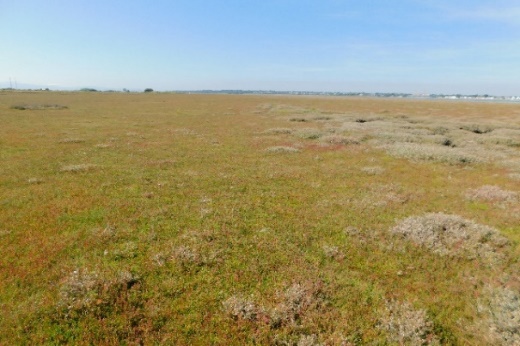  **b** |
| --- |


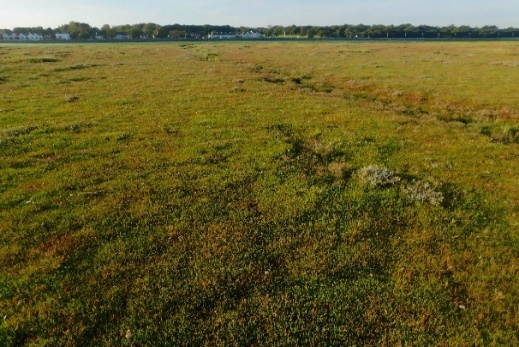


**a**

Figure S1: Images showing a) upper salt marsh zone H, predominantly Salicornia Sp. with a mix of meadow flowers and grasses. In some areas within the upper marsh zones, pockmarks can be seen as barren, muddy depressions, sometimes containing a layer of water and dried layers of detritus OM.

**a**

**b**

Figure S2: Images of mid-low marsh zone M, displaying the increased diversity of vegetation with areas of sparsity, channelling and highly heterogeneous regions a) South lagoon salt marsh and b) North lagoon salt marsh.


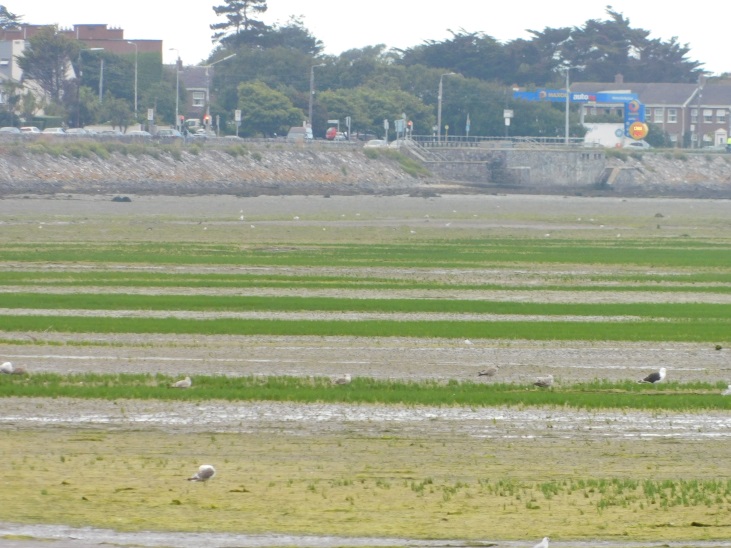

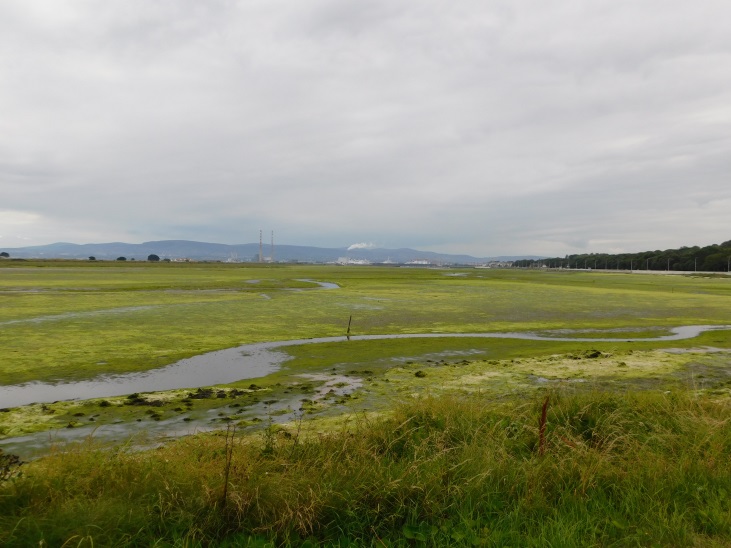


**b**

**a**

Figure S3: Tidal zone T in Bull Island’s lagoons displaying marine characteristics with high algae growth, seaweed, sandy patches and a lack of vegetation. a) Riverine inputs influence the degree of algae and seaweed blooms seen here in the South lagoon and, b) the emergence and lateral expansion of *Salicornia* flats in the inner North lagoon.

**Polyaromatic Hydrocarbon (PAH) Extraction**

Samples were air-dried in the dark for 36-96hrs (dependant on % organic matter), screened, homogenised and sieved to 420 **µ**m particle size prior to extraction. The mass of sample chosen for extraction was dependant on the % OM content of individual samples. Sample weights between 3-5 g were suitable for higher OM samples (≥20% OM) and 5-7 g of sample was extracted for samples of lower OM content (≤20% OM). Final quantification was standardised to original sample mass used. All samples were extracted in triplicate.

Sediment samples were extracted using a Dionex Accelerated Solvent Extractor (ASE) (ASE® 200 Accelerated Solvent Extractor) instrument. A known mass of sample was briefly mixed with pre-furnaced sand and sodium sulfate before packing into 33ml stainless steel extraction cells. Cells were assembled by capping one end (threaded steel cap with a Teflon lined opening for needle entry) and placing in a new cellulose filter disc. The sample was funnelled into the tube and a second filter was placed on top before sealing with an end cap and placing on the sample carousel. Dichloromethane (DCM) was injected into the extraction cell with subsequent heating to 100°C and a hold time of 6 mins. High purity nitrogen was then passed through the extraction cell at a pressure of 1500psi, with subsequent collection of extractant solvent underneath in airtight and sterilised amber collection vials. Solvent entered the vials through a needle after piercing a new PTFE lined septum for every vial to reduce chances of cross contamination. The process included a 60 % flush volume and a last purge of 60 seconds. Each extraction cell was extracted for 1 full cycle and allowed to cool to room temperature. Sample extracts were reduced down (~500ul) at room temperature/low vacuum using rotary evaporation and made up to 1 ml in solvent washed round-bottom flasks. Finally, the 1 ml samples were transferred into pre-labelled GC vials and a spatula tip full of activated copper was added to the extracts to remove sulfur**.** The 1 ml extracts were shaken for 24 hours in the dark and were then subsampled and stored upright at -30 °C until analysis.

**Analysis of PAHs**

Analysis of extracts was carried out on an Agilent 7890N gas chromatograph coupled to an Agilent 5973N mass selective detector operating in electron impact mode at 70 eV. The column was a 30 m HP-5MS column (0.25 mm i.d., 1 μm film thickness). The GC-Ms interface and ion source were set at 300⁰C and 250⁰C respectively. Selected ion mode (SIM) was used for analysis. The GC-MS method was set up as follows; the column flow rate was set to 1ml/min, injection volume of 1 μl with a split ratio of 2:1 was set in conjunction with an injection port split liner. A solvent delay of 6 mins was integrated into the method. The initial oven temperature was 70 °C for 0.5 min and increased at 10 °C/min to 300 °C and held for 20min; with a total run time of 45 min. The data was processed using Chemstation software, combining mass spectral library databases (NIST and Wiley), certified PAH standards, spectra interpretation, retention times and referenced literature to confirm presence of identified compounds. An internal standard of 100ppm 5α-cholestane was used for all extracts and blanks. 16 priority PAHs were quantified in SIM mode using the cholestane internal standard and a calibration curve produced from a 16 PAH certified reference material (CrM) standard. The limit of quantification (LOQ) and limit of detection (LOD) was calculated for each group of PAH compounds according to the number of benzene rings. A LOQ and LOD was determined for 2 ring PAHs using naphthalene, fluorene (3- ring), phenanthrene (4-ring), pyrene (5-ring) and benzo (ghi) perylene (6-ring). The LOQ for each PAH ranged from 22.50 ng/g (all 5 and 6-ring PAHs) to 67.50 ng/g (fluorene). The LOD ranged from 7.43 ng/g (all 5 and 6-ring PAHs) to 22.20 ng/g (fluorene).

A % recovery study was carried out using the described ASE method. Previously tested, PAH free sediment samples were spiked with the following deuterated PAH standards; naphthalene (d8), acenaphthene (d10), anthracene and perylene (d12) giving a recovery range of 75 - 100 %. recovery was determined using a range of sediment types (sand, mud and sandy mud) to represent the heterogeneity of samples from Bull Island. The value for the sum of 16 priority PAHs at individual sample sites and subsequent zones was used to assign a PAH pollutant level classification as previously suggested by Baumard et al as (a) low, 0–100 ng/g; (b) moderate, 100–1,000 ng/g; (c) high, 1,000–5,000 ng/g; and (d) very high, >5,000 ng/g.

Table S1.a: Table displaying the mean (upper ‘**bold’** figure) and standard deviation (lower ‘*Italic’* figure) of measurements attained for abiotic variables (n= 3, except for Clay, Silt and Sand, where n=1 analytical replicate from a bulk composite) at individual sample sites across salt marsh sediments.

Table S1. b: Table displaying the mean (upper ‘**bold’** figure) and standard deviation (lower ‘*Italic’* figure) of measurements attained for abiotic variables (n= 3, except for Clay, Silt and Sand, where n=1 analytical replicate from a bulk composite) at individual sample sites across intertidal sediments.

Table S2.a: Spearman’s correlation results displaying r values, where significant values (p<0.05) are indicated by **bold** text.

Table S2.b: Spearman’s correlation results displaying calculated significant values for table S2. a [ (α=0.05) indicated by b**old** text], generated for all variables measured across all sample sites (n=39).

Table S.3: Displaying significant results for Mann-Whitney testing of geochemical variable measurements between NL and SL grouped samples.
